# The association between lutein and zeaxanthin intake and multi-level biological aging

**DOI:** 10.3389/fnut.2025.1618158

**Published:** 2025-08-08

**Authors:** Meiyi Tao, Lin Zhang, Caidi Jiang, Jia Xiang, Shipeng Chen, Songwen Tan, Shengli Sun

**Affiliations:** ^1^Hunan Provincial People's Hospital (First Affiliated Hospital of Hunan Normal University), Changsha, China; ^2^Department of Epidemiology and Preventive Medicine, The School of Public Health and Preventive Medicine, Monash University, Melbourne, VIC, Australia; ^3^Suzhou Industrial Park Monash Research Institute of Science and Technology, Suzhou, China; ^4^Xiangya School of Pharmaceutical Sciences, Central South University, Changsha, China; ^5^Hunan Pilot Free Trade Zone Global Cell Bank, Changsha, China; ^6^Hunan Provincial Key Laboratory of the Research and Development of Novel Pharmaceutical Preparations, Changsha Medical University, Changsha, China; ^7^Monash University-Southeast University Joint Research Institute, Suzhou, China

**Keywords:** biological aging, lutein, zeaxanthin, carotenoid, NHANES

## Abstract

**Objective:**

This study investigates the potential association between lutein and zeaxanthin (LZ) intake, particularly lutein, and biological aging. The study aims to explore how LZ intake affects the biological aging progression, particularly in organs like the liver, kidneys, and cardiovascular system, and explore the potential mechanism of lutein as the main carotenoid mediating this effect.

**Methods:**

We analyzed biological aging using biological age calculations based on data from the NHANES 2007–2015 database. Various adjusted models were used to assess the relationship between LZ intake and aging phenotypes. Transcriptome analysis was conducted to explore the potential mechanisms underlying the anti-aging effects of lutein.

**Results:**

A higher intake of LZ was associated with a slower biological aging rate (*P* < 0.01), observed in major organs such as the liver and kidneys, as well as the cardiovascular system. LZ intake showed a significant negative correlation with biological aging acceleration (*P* < 0.05). Enrichment analysis suggested that lutein's anti-aging effects might be mediated through telomere regulation and modulation of aging-related metabolic pathways. Additionally, lutein intake appeared to reduce pro-inflammatory Th1 cell abundance, further suggesting a potential anti-aging effect by suppressing inflammation. Sustained lutein intake also led to a decrease in the expression of aging phenotype-related molecules. However, in the evaluation of linear relationships, excessive lutein intake beyond a certain threshold may not yield additional benefits.

**Conclusion:**

Combined LZ intake is associated with attenuated multi-level biological aging [OR (95% CI): 0.93 (0.88, 0.93), *P* = 0.016] and high LZ intake significantly reduce the risk of all-cause death (*P* < 0.001), with lutein driving systemic effects via telomere regulation and inflammation suppression. These findings highlight lutein's translatable potential for aging interventions and provide insights for dietary strategies in aging health management.

## Graphical Abstract

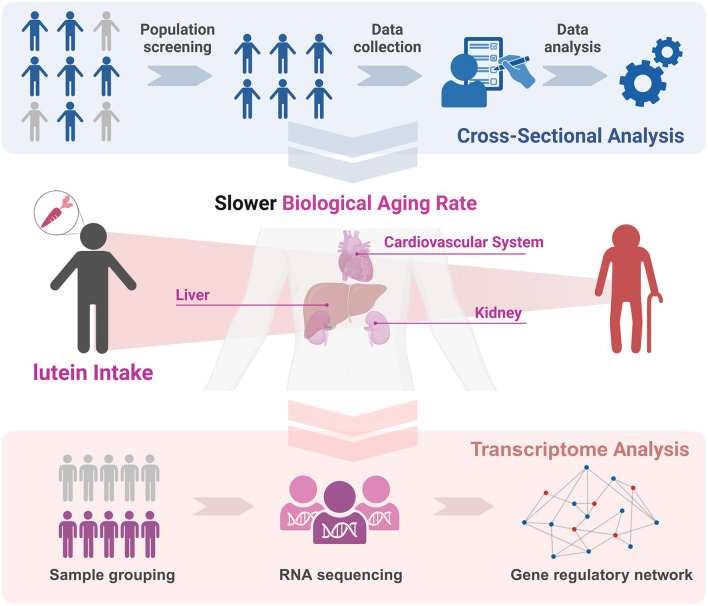

## 1 Introduction

Aging, as a Multisystem Phenomenon, is a primary risk factor for chronic diseases and mortality (1), characterized by progressive functional decline across multiple organ systems. Crucially, biological aging exhibits significant inter-organ heterogeneity—while chronological age (CA) advances uniformly, organs like the liver (detoxification hub), kidneys (metabolic waste clearance), and cardiovascular system (systemic perfusion) experience divergent aging trajectories that collectively determine organismal health span (2). This multisystem perspective necessitates organ-specific biological aging quantification to identify targeted interventions (3–5).

Since the inception of biological age (BA) calculation (2), BA estimation has evolved from CA-biomarker correlations (6) to algorithms treating CA as an integrated biomarker (5). The Klemera-Doubal Method (KDM) now demonstrates superior mortality prediction (3, 7) by incorporating organ-specific biomarkers.

Among modifiable aging factors, as multiorgan anti-aging agents, carotenoids - particularly lutein and zeaxanthin (LZ)—show exceptional promise (8). While renowned for retinal protection (9), their systemic anti-aging potential remains underexplored. Mechanistically, LZ could scavenge lipid peroxides in hepatic/kidney tissues (9–11), protect against skin damage induced by free radicals (12), and these substances could be considered “longevity vitamins” (9).

Although there are relevant evidences that LZ intake has certain anti-inflammatory and anti-aging effects, there are few studies to quantify the association between LZ and human organ-specific biological aging.

Leveraging NHANES (National Health and Nutrition Examination Survey) database, we constructed biological aging acceleration models for various organs and systems (liver/kidney/cardiovascular) to assess LZ intake associations with organ-specific and systemic aging. Furthermore, we also analyzed the potential effects and the specific mechanisms of sustained high concentrations of lutein on aging phenotypes via transcriptomics. This multi-level analysis provides actionable insights for dietary strategies against heterogeneous aging (Graphical Abstract).

## 2 Methods

### 2.1 Study design and population

**Figure 1:**
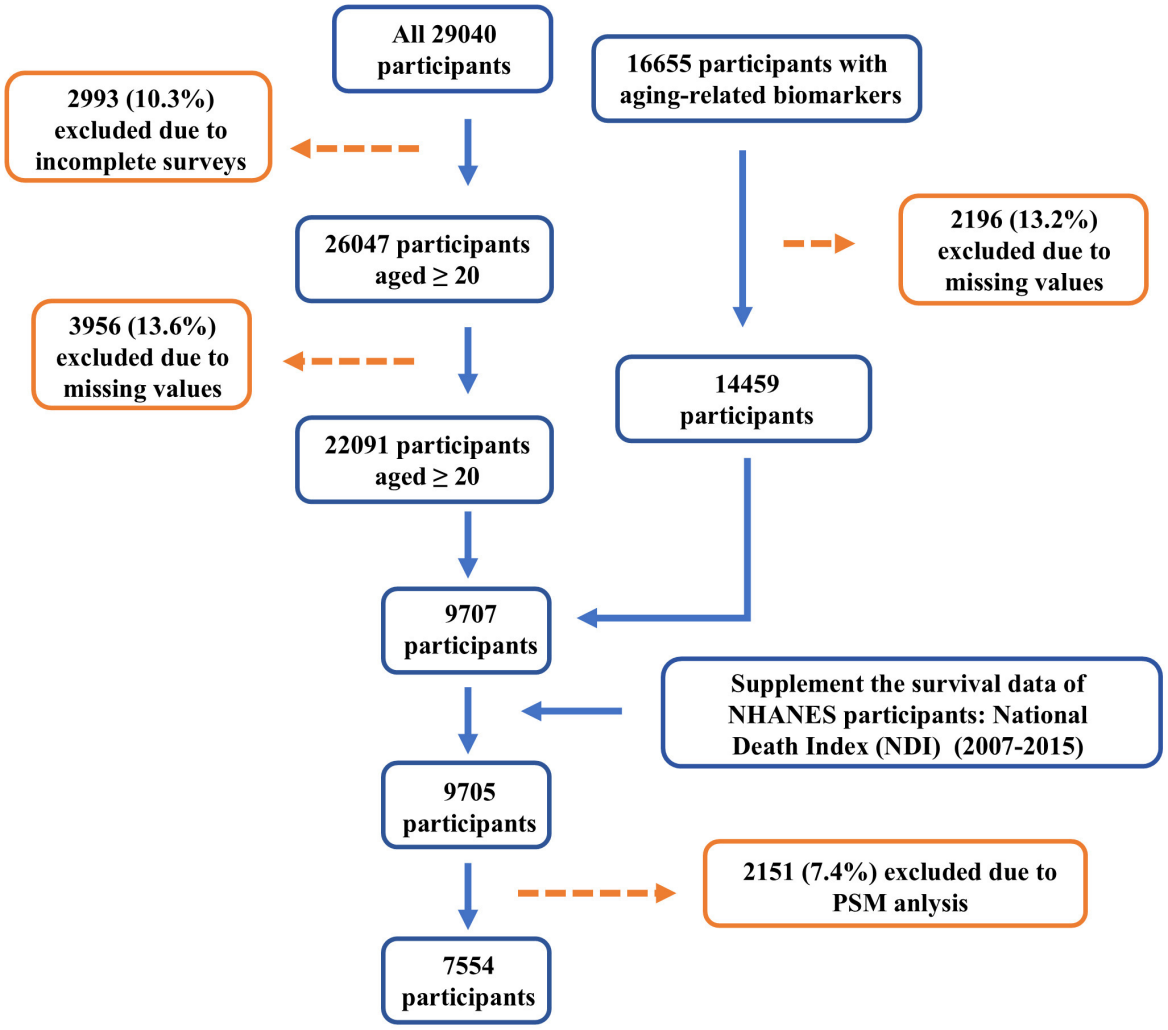
Flow chart of study population.

This study utilized data from the National Health and Nutrition Examination Survey (NHANES), a program conducted in the United States to assess the health and nutritional status of both adults and children (https://www.cdc.gov/nchs/nhanes/). NHANES is a cross-sectional survey based on the general U.S. population. Data from NHANES 2007–2015 were analyzed, and 29,040 participants were initially selected, all of whom provided written informed consent. The focus was primarily on adults aged 20 and above. After combining the databases using unique identifiers for each participant, cases with missing data on physical examinations, alcohol consumption, smoking, and carotenoid intake (19,335 cases) were excluded. Ultimately, 7,554 participants were included in the study (Figure 1).

### 2.2 Assessment of biological age

Biological age was assessed at three different levels to quantify the degree of biological aging, including cardiovascular biological age (BaC), liver biological age (BaL), and kidney biological age (BaK). The biomarkers related to BaC were collected, including triglycerides, high-density lipoprotein, low-density lipoprotein, fasting blood glucose, systolic blood pressure, diastolic blood pressure, and total cholesterol. The biomarkers related to BaL included gamma-glutamyl transferase, serum aspartate aminotransferase, serum alanine aminotransferase, and albumin, because of the low organ specificity, ALP elevation may also occur in bone diseases (13), placental diseases and intestinal diseases (14), it was not included in the evaluation of liver biological age. The biomarkers for BaK included serum creatinine and serum uric acid. The biological age of different organs was calculated using the biological age algorithm KDM (15, 16). The ratio of biological age to chronological age (BA ratio) was used as an indicator for evaluating biological aging. Additionally, the difference between biological age and chronological age (BA acceleration) was used as a measure of aging acceleration.

### 2.3 Lutein and zeaxanthin intake

Lutein and zeaxanthin (LZ), as carotenoids in the diet, play an important role in maintaining human health. Based on the variables collected in the NHANES database, we included both dietary and supplement sources of LZ and calculated the total intake of LZ from both sources. The intake of LZ from both sources was measured over 2 days, and the average intake of the 2 days was used as the final LZ intake value.

### 2.4 Smoking exposure

The following variables were collected: SMQ020, SMQ040, SMQ050Q, SMQ050U. Smoking status was classified into three categories based on the information from these variables: Current smoker, Former smoker, and Never smoker. Individuals who had smoked fewer than 100 cigarettes in their lifetime were considered as “Never smokers,” while those who had stopped smoking for more than 1 month were classified as “Former smokers.”

### 2.5 Body mass index (BMI)

BMI was categorized into the following groups based on BMI values: “Underweight (< 18.5),” “Normal (18.5 to < 25),” “Overweight (25 to < 30),” “Obese (30 or greater).”

### 2.6 Total calorie intake

Total calorie intake was obtained from the variables collected in the NHANES database, including intake data from both the first and second days. If data from the second day were available, the average intake from both days was used; otherwise, only the intake from the first day was considered.

### 2.7 Alcohol consumption history

The following variables were collected: “ALQ11,” “ALQ10,” “ALQ120Q,” “ALQ120U.” The alcohol consumption was classified into four categories: “Non-drinker,” “ < 1 drink/month,” “1–10 drinks/month,” and “10+ drinks/month.” Participants who had consumed fewer than 12 alcoholic drinks in their lifetime were categorized as “Non-drinker.”

### 2.8 Other covariates

We included additional covariates based on previous studies. Participants' demographic information, including gender, age, race (Mexican American, Other Hispanic, Non-Hispanic White, Non-Hispanic Black, and Other races), education level (less than 9th grade, 9-11th grade, high school graduate, some college/AA degree, and college graduate), and household income, were collected via questionnaires. These factors may have potential associations with biological aging or dietary carotenoid intake.

### 2.9 Survival data

Survival data were linked to the National Death Index (NDI), and mortality follow-up data for NHANES participants from 2007 to 2015 were incorporated to construct Cox proportional hazards models to evaluate the effect of lutein intake on all-cause mortality.

### 2.10 PSM adjustment

The processed data were further adjusted using Propensity Score Matching (PSM) to account for potential confounders, including age, gender, race, education level, and income.

### 2.11 Data acquisition

Data mining and analysis were performed using R version 4.4.0. Microarray datasets from Gene Expression Omnibus (GEO, https://www.ncbi.nlm.nih.gov/geo/) were retrieved using the R package “GEOquery” (17). Specifically, we used the dataset GSE151683 (18), which includes transcriptomic data related to high lutein and high lycopene intake. In this study, we focused on the high lutein intake group and the low lutein control group, and differential gene expression was analyzed using the “limma” package (19) to identify fold changes between groups. The differentially expressed genes were then subjected to subsequent analysis.

### 2.12 Enrichment analysis

Biological process enrichment analysis was performed using the Gene Prioritization by Evidence Counting (GPEC) algorithm from Metascape (http://metascape.org/), with the inclusion criteria of *P* value < 0.01, a minimum count of 3, and an enrichment factor >1.5. Clustering of the entry nodes was conducted based on the similarity of the members. Gene set enrichment analysis (GSEA) was conducted using the R package “clusterProfiler” (20) to assess changes in biological functions following sustained high lutein intake. The background gene sets (C2: curated gene sets, C5: ontology gene sets) were provided by the Molecular Signatures Database (MSIGDB) (https://www.gsea-msigdb.org/gsea/index.jsp). Metabolic pathway enrichment was performed using the R package “IOBR” (21), integrating three algorithms (ssGSEA, PCA, and z-score) to quantify the metabolic enrichment level of each sample. Differential scoring analysis was then performed using “limma” based on *t*-values to evaluate metabolic differences between groups.

### 2.13 Immune scoring

Immune scores for different samples were calculated using the immune algorithms (xCell, MCPcounter) from the R package “IOBR” to quantify differences in the abundance of various immune cell types in peripheral blood after sustained high lutein intake between different groups. MCP-counter and xCell algorithms quantified the abundance of immune cells between clusters based on marker gene expression. Only immune cell types showing statistically significant differences (*P* < 0.05) between groups were reported.

### 2.14 Statistical analysis

All analyses were performed using R software version 4.4.0. Non-linear relationships between lutein intake and biological age acceleration across different patterns were tested using restricted cubic splines (RCS) implemented in the “rms” R package. Continuous variables were expressed as interquartile ranges (IQR), and categorical variables were expressed as counts (*n*) and percentages (%). Multivariate logistic regression models and multivariate Cox regression models were used to analyze the relationship between lutein intake and biological age acceleration across different patterns, as well as the association with all-cause mortality. Lutein intake levels were categorized into quartiles (Q1, Q2, Q3, and Q4) with Q1 as the reference group, and the odds ratios (ORs) and 95% confidence intervals (95% CIs) were used to describe the relationship between lutein intake and biological aging. Further non-linear relationship tests between lutein intake and biological age acceleration were conducted using RCS with the “rms” R package. Demographic differences in carotenoid intake levels, including age, gender, race, education, smoking, alcohol intake, ratio of family income to poverty (PIR), BMI, waist circumference, total calorie intake, and different sources of LZ intake, were compared using the Kruskal-Wallis test. Interaction analysis was performed using the likelihood ratio test. Paired differences between groups were compared using two-tailed paired Student's *t*-test. A *P* value of < 0.05 was considered statistically significant for all analyses.

## 3 Results

### 3.1 Baseline characteristics

In this study, after applying the inclusion and exclusion criteria, we initially included 9,705 participants (Figure 1). We divided the participants into four groups (Q1–Q4) based on their total LZ intake, ranging from low to high. The Q1 group [0.32 (0.21, 0.41)] had the lowest LZ intake level, while the Q4 group [3.04 (2.19, 5.10)] had the highest. Baseline characteristics showed significant differences in the distribution of most covariates across the four groups (Supplementary Table S1). To reduce the confounding effect of covariates on the epidemiological outcome variables, we performed PSM to adjust for data distribution. After PSM adjustment, 7,554 participants were included in the final analysis (Table 1).

**Table 1:** Baseline characteristics of participants under different L/Z total intake levels.

**Characteristic**	**Q1 *N* = 1,897 (25%)^1^**	**Q2 *N* = 1,897 (25%)^1^**	**Q3 *N* = 1,896 (25%)^1^**	**Q4 *N* = 1,896 (25%)^1^**	***P* value^2^**
**Age.group**					0.11
20–39 years	711 (37%)	606 (32%)	616 (32%)	625 (33%)	
40–59 years	707 (37%)	717 (38%)	743 (39%)	752 (40%)	
≥60 years	479 (25%)	574 (30%)	537 (28%)	519 (27%)	
**Sex**					**0.002**
Female	967 (51%)	989 (52%)	895 (47%)	1,071 (56%)	
Male	930 (49%)	908 (48%)	1,001 (53%)	825 (44%)	
**Race**					0.1
Non-Hispanic White	1,319 (70%)	1,363 (72%)	1,313 (69%)	1,369 (72%)	
Non-Hispanic Black	207 (11%)	158 (8.3%)	181 (9.5%)	198 (10%)	
Mexican American	154 (8.1%)	148 (7.8%)	174 (9.2%)	116 (6.1%)	
Other Hispanic	99 (5.2%)	101 (5.3%)	101 (5.3%)	95 (5.0%)	
Other/multiracial	118 (6.2%)	128 (6.8%)	127 (6.7%)	117 (6.2%)	
**Education.attainment**					**< 0.001**
Less than 9th Grade	113 (5.9%)	68 (3.6%)	104 (5.5%)	63 (3.3%)	
9-11th Grade	213 (11%)	190 (10%)	229 (12%)	154 (8.1%)	
High School Grad/GED	444 (23%)	389 (21%)	497 (26%)	372 (20%)	
Some college or AA degree	656 (35%)	639 (34%)	647 (34%)	636 (34%)	
College graduate or above	470 (25%)	610 (32%)	419 (22%)	671 (35%)	
**BMI.group**					0.3
Underweight (< 18.5)	32 (1.7%)	25 (1.3%)	30 (1.6%)	27 (1.4%)	
Normal (18.5 to < 25)	512 (27%)	484 (26%)	519 (27%)	602 (32%)	
Overweight (25 to < 30)	646 (34%)	626 (33%)	635 (33%)	605 (32%)	
Obese (30 or greater)	707 (37%)	762 (40%)	712 (38%)	662 (35%)	
**Alq.group**					0.6
Non-drinker	430 (23%)	411 (22%)	430 (23%)	430 (23%)	
< 1 drinks/month	408 (22%)	387 (20%)	453 (24%)	373 (20%)	
1-10 drinks/month	746 (39%)	757 (40%)	698 (37%)	762 (40%)	
>10 drinks/month	313 (17%)	343 (18%)	314 (17%)	330 (17%)	
**Smoke.group**					0.14
Current smoker	582 (31%)	514 (27%)	568 (30%)	484 (26%)	
Former smoker	287 (15%)	338 (18%)	324 (17%)	331 (17%)	
Never smoker	1,028 (54%)	1,046 (55%)	1,004 (53%)	1,081 (57%)	
Age	46 (32, 60)	49 (36, 62)	49 (35, 62)	49 (34, 60)	**0.004**
PIR	2.88 (1.29, 4.64)	3.13 (1.61, 5.00)	2.73 (1.44, 4.76)	3.30 (1.64, 5.00)	**< 0.001**
BMI	28 (24, 33)	28 (25, 33)	28 (24, 32)	27 (24, 32)	0.066
Waist	99 (88, 111)	100 (89, 110)	98 (88, 110)	97 (86, 108)	**0.048**
Total calories	1,754 (1,348, 2,276)	1,983 (1,550, 2,462)	2,127 (1,647, 2,749)	2,010 (1,588, 2,641)	**< 0.001**
Dietary.LZ	0.32 (0.20, 0.41)	0.67 (0.56, 0.77)	1.15 (0.98, 1.36)	2.83 (2.06, 4.56)	**< 0.001**
Supplement.LZ	0.25 (0.25, 0.25)	0.25 (0.25, 0.25)	0.25 (0.25, 0.30)	0.30 (0.25, 1.34)	**< 0.001**
Total.LZ	0.32 (0.21, 0.41)	0.69 (0.60, 0.78)	1.20 (1.02, 1.39)	3.04 (2.19, 5.10)	**< 0.001**

^1^*n* (%); Median (Q1, Q3).

^2^Pearson's X^2^: Rao & Scott adjustment; Design-based KruskalWallis test.

*P* < 0.05 is marked with bold values.

Adjusted baseline data indicated significant differences in both dietary and supplement sources of LZ intake across the four groups (*P* < 0.001). There were also significant differences in the distribution of gender (*P* = 0.002), age (*P* = 0.004), education level (*P* < 0.001), income (*P* < 0.001), total calorie intake (*P* < 0.001), and waist circumference (*P* = 0.048) across the different lutein intake groups (Table 1). In terms of education level, the highest intake group (Q4) had a larger proportion of participants with higher education (*P* < 0.001). Among the Q4 group, 35% had a college degree or higher, while only 3.3% had less than a 9th-grade education. The Q4 group also had the highest income level [3.30 (1.64, 5.00)]. Additionally, the total calorie intake in Q3 [2,127 (1,647, 2,749)] and Q4 [2,010 (1,588, 2,641)] was relatively higher.

### 3.2 Association between carotenoid intake and biological aging progression

To evaluate whether LZ intake influences the progression of biological aging, we calculated the biological age at various levels (cardiovascular, kidney, liver, and overall) for participants and assessed the aging trend using biological age ratios.

First, we evaluated the distribution of biological age ratios for different levels of dietary LZ intake (Supplementary Table S2). As LZ intake increased, the biological age ratios for different organ systems (cardiovascular, kidney, liver, and overall) significantly decreased (pre-PSM adjustment: *P* < 0.05). After adjusting for covariates using PSM, although the median values for all four biological age ratios decreased, only the overall biological age ratio showed a significant difference (*P* = 0.025), with the ratios for Q1 and Q4 being 0.99 (0.84, 1.16) and 0.95 (0.82, 1.13), respectively. Next, we evaluated the distribution of biological age ratios at different total LZ intake levels. As total LZ intake increased (Table 2), the biological age ratios for cardiovascular, kidney, liver, and overall aging significantly decreased (before PSM adjustment: *P* < 0.001 or *P* = 0.001). After PSM adjustment, significant differences were still observed in three biological age ratios (kidney, liver, and overall), with median values for organ and overall biological age ratios in the high LZ intake groups (Q3 and Q4) remaining below 1, indicating that LZ intake from multiple sources collectively influences the biological aging process.

**Table 2:** Characteristics of biological age acceleration under different L/Z total intake before and after the treatment of properness score matching.

**Total L/Z Intake**	**Q1^1^**	**Q2^1^**	**Q3^1^**	**Q4^1^**	***P* value^2^**
**Before propensity score matching**					
Total.LZ	0.30 (0.19, 0.39)	0.67 (0.57, 0.78)	1.23 (1.04, 1.46)	3.27 (2.33, 5.68)	**< 0.001**
Dietary.LZ	0.30 (0.18, 0.38)	0.65 (0.55, 0.76)	1.18 (0.99, 1.42)	2.95 (2.14, 4.89)	**< 0.001**
Supplement.LZ	0.25 (0.25, 0.25)	0.25 (0.25, 0.25)	0.25 (0.25, 0.30)	0.30 (0.25, 2.00)	**< 0.001**
BA_ratio.Cardio	0.99 (0.83, 1.21)	0.99 (0.81, 1.20)	0.96 (0.79, 1.17)	0.95 (0.79, 1.12)	**< 0.001**
BA_ratio.Kidney	1.00 (0.88, 1.17)	1.00 (0.88, 1.14)	0.98 (0.87, 1.11)	0.98 (0.86, 1.11)	**0.001**
BA_ratio.Liver	0.89 (0.55, 1.22)	0.85 (0.55, 1.18)	0.85 (0.59, 1.15)	0.79 (0.54, 1.08)	**< 0.001**
BA_ratio.Total	1.00 (0.84, 1.17)	0.98 (0.84, 1.15)	0.96 (0.82, 1.13)	0.94 (0.81, 1.11)	**< 0.001**
**After propensity score matching**					
Total.LZ	0.32 (0.21, 0.41)	0.69 (0.60, 0.78)	1.20 (1.02, 1.39)	3.04 (2.19, 5.10)	**< 0.001**
Dietary.LZ	0.32 (0.20, 0.41)	0.67 (0.56, 0.77)	1.15 (0.98, 1.36)	2.83 (2.06, 4.56)	**< 0.001**
Supplement.LZ	0.25 (0.25, 0.25)	0.25 (0.25, 0.25)	0.25 (0.25, 0.30)	0.30 (0.25, 1.34)	**< 0.001**
BA_ratio.Cardio	0.98 (0.83, 1.20)	0.99 (0.82, 1.19)	0.98 (0.80, 1.19)	0.96 (0.80, 1.14)	0.058
BA_ratio.Kidney	1.00 (0.89, 1.16)	1.00 (0.88, 1.13)	0.98 (0.88, 1.12)	0.98 (0.86, 1.13)	**0.029**
BA_ratio.Liver	0.88 (0.56, 1.19)	0.84 (0.55, 1.14)	0.86 (0.59, 1.18)	0.81 (0.54, 1.12)	**0.044**
BA_ratio.Total	0.99 (0.84, 1.16)	0.98 (0.84, 1.14)	0.98 (0.82, 1.14)	0.94 (0.81, 1.12)	**0.007**

^1^Median (Q1, Q3).

^2^Design-based KruskalWallis test.

*P* < 0.05 is marked with bold values.

**Figure 2:**
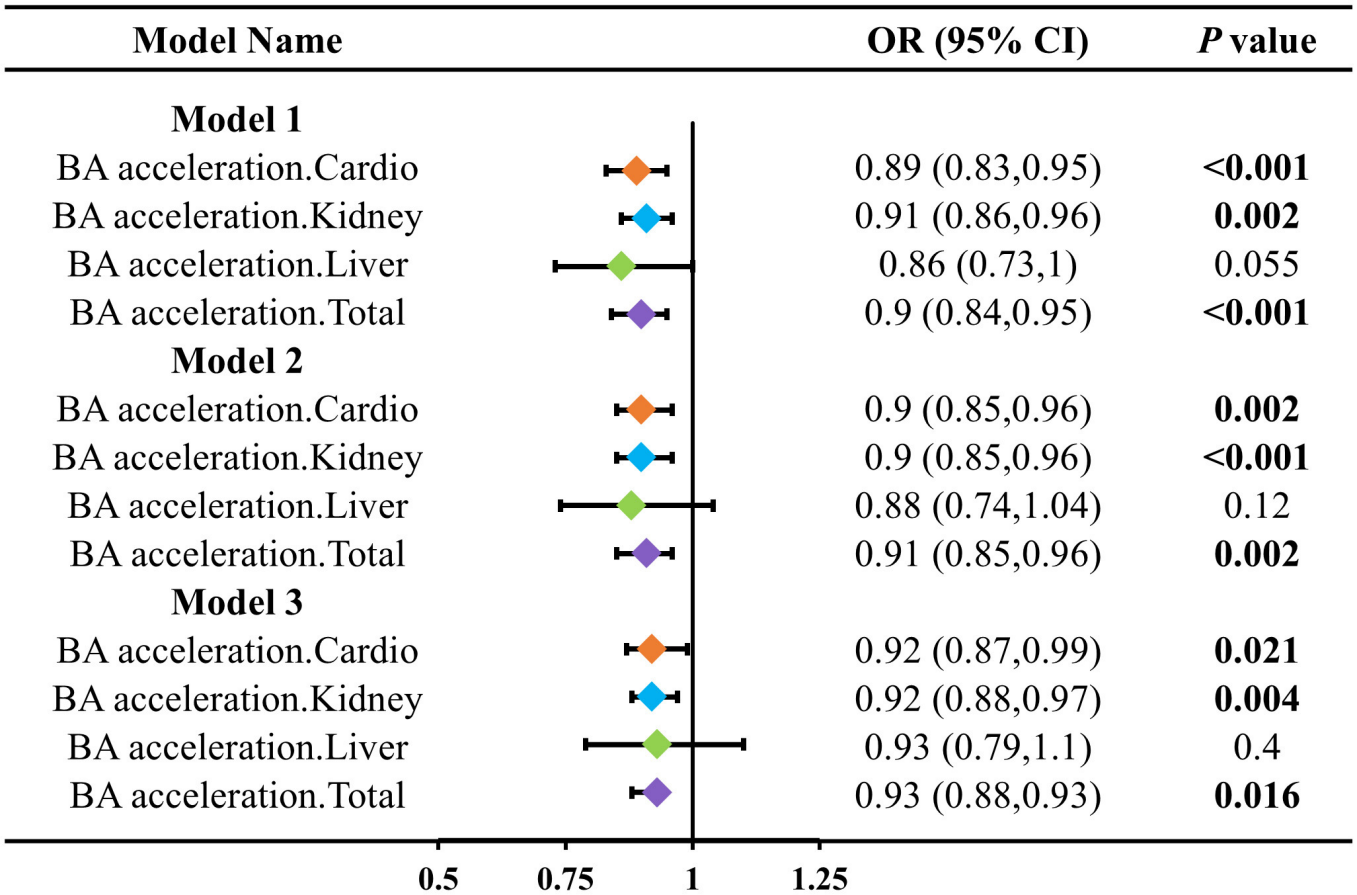
Association between LZ total intake levels and different patterns of biological age acceleration. Model 1 adjusted for age; Model 2 adjusted for model 1, and gender, race, education level, income; Model 3 adjusted for model 2, and smoking, alcohol intake, BMI, total calories.

To further assess the impact of LZ intake on the acceleration of biological aging in different organ systems, we calculated the biological age acceleration for four types (cardiovascular, kidney, liver, and overall) and constructed three models adjusted for different covariates (Figure 2). The results showed that total LZ intake significantly reduced the biological age acceleration in various organs (cardiovascular, kidney). Cardiovascular aging acceleration decreased by 0.89 (95% CI: 0.83–0.95), 0.90 (95% CI: 0.85–0.96), and 0.92 (95% CI: 0.87–0.99) in the three models, respectively. Kidney aging acceleration decreased by 0.91 (95% CI: 0.86–0.96), 0.90 (95% CI: 0.85–0.96), and 0.92 (95% CI: 0.88–0.97), respectively. Total LZ intake also reduced the overall aging acceleration in all three models by 0.90 (95% CI: 0.84–0.95), 0.91 (95% CI: 0.85–0.96), and 0.93 (95% CI: 0.88–0.93), respectively. After adjusting for covariates, the effect of total LZ intake on liver biological age acceleration was attenuated.

**Figure 3:**
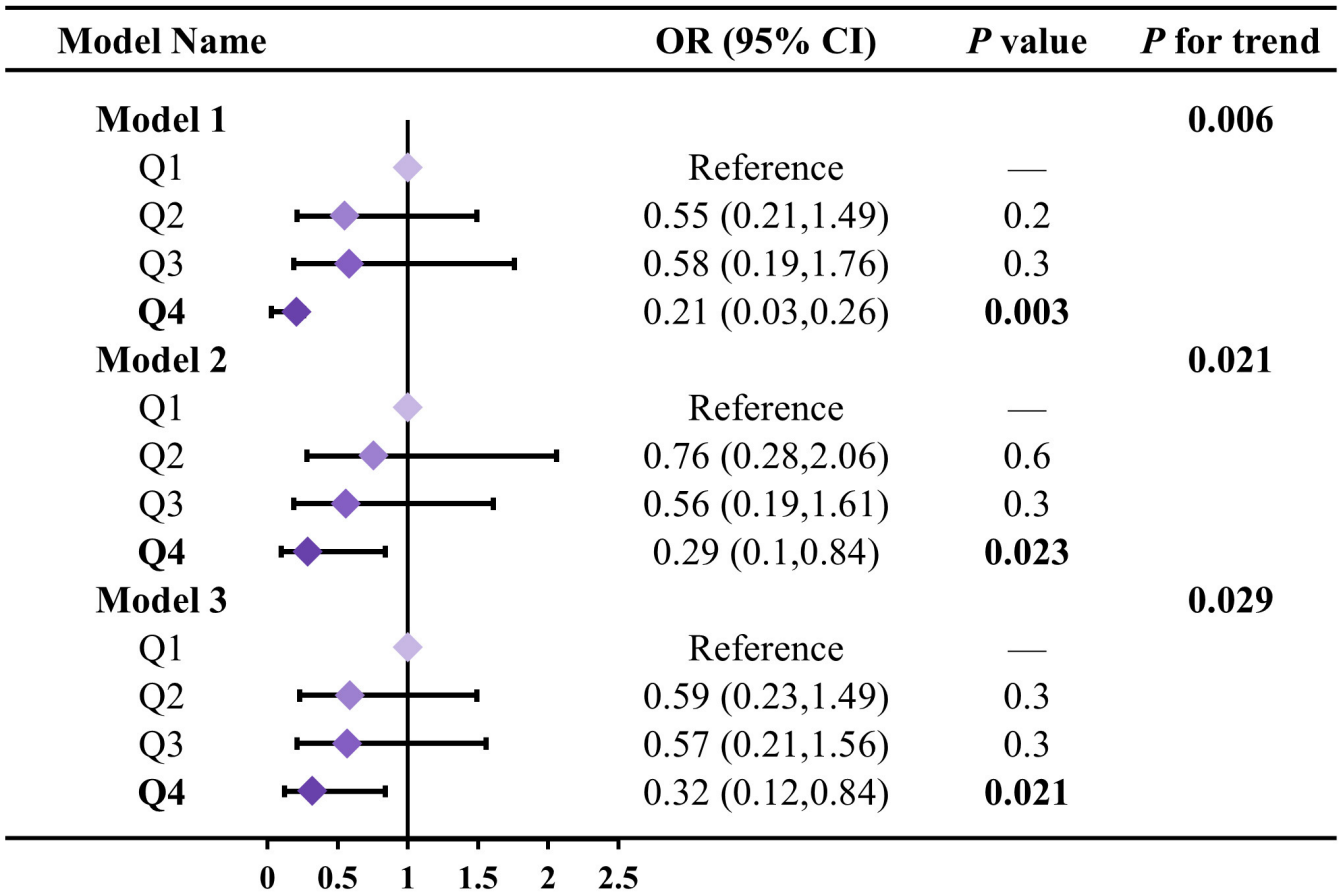
Association between LZ total intake levels and total biological age acceleration. Model 1 adjusted for age; Model 2 adjusted for model 1, and gender, race, education level, income; Model 3 adjusted for model 2, and smoking, alcohol intake, BMI, total calories. Test for trend based on variable containing median value for each quartile.

We further evaluated the differential effects of LZ intake on biological aging acceleration at different intake levels (Figure 3). In three models measuring overall biological age acceleration, compared to the Q1 group, the biological age acceleration in the Q4 group was reduced by 0.21 (95% CI: 0.03–0.26), 0.29 (95% CI: 0.10–0.84), and 0.32 (95% CI: 0.12–0.84), with significant trend tests in all three models (*P* for trend: 0.006; 0.021; 0.029). Additionally, we assessed the differential effects of LZ intake on the biological aging acceleration of other organs at different intake levels (Supplementary Table S3). For kidney and liver biological aging acceleration, high LZ intake (Q4) showed more significant differences in the effect on aging acceleration compared to low LZ intake (Q1) (all *P* < 0.05). For kidney and liver biological aging acceleration, the trend tests for the different LZ intake levels were also more significant (all *P* < 0.05).

**Figure 4:**
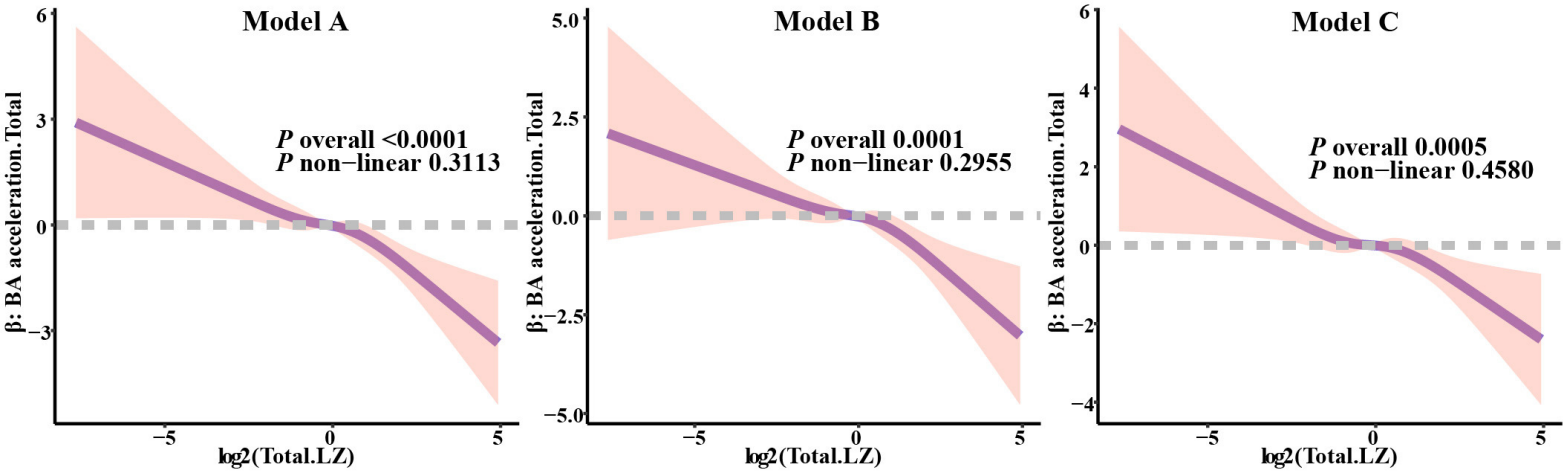
Analysis of restricted cubic spline regression between total LZ intake levels and total biological age acceleration. Model 1 adjusted for age; Model 2 adjusted for model 1, and gender, race, education level, income; Model 3 adjusted for model 2, and smoking, alcohol intake, BMI, total calories.

To further assess the linear relationship between total LZ intake and biological age acceleration, we constructed three models of Restricted Cubic Splines (RCS) for the evaluation of biological aging acceleration across different patterns. For overall biological aging acceleration (Figure 4), in Model 1 (*P* overall < 0.0001), Model 2 (*P* overall = 0.0001), and Model 3 (*P* overall = 0.0005), the correlation between the biological aging acceleration beta values and the transformed total LZ intake was significant, with no significant non-linear relationship observed. Additionally, in the evaluation of biological aging acceleration in different organs (Supplementary Figure S1), after multiple adjustments in the models, significant correlations were still evident (Model 3_Cardio, *P* = 0.0241; Model 3_Kidney, *P* = 0.0162; Model 3_Liver, *P* = 0.0021). Although none of the models for biological aging acceleration in the three organs exhibited significant non-linear trends, in the liver biological aging acceleration assessment, we observed that continuous increases in LZ intake did not lead to a substantial reduction in the model beta values. This suggests that total LZ intake can significantly reduce the risk of biological aging acceleration within a certain range, while excessive intake may not effectively further delay biological aging acceleration.

**Figure 5:**
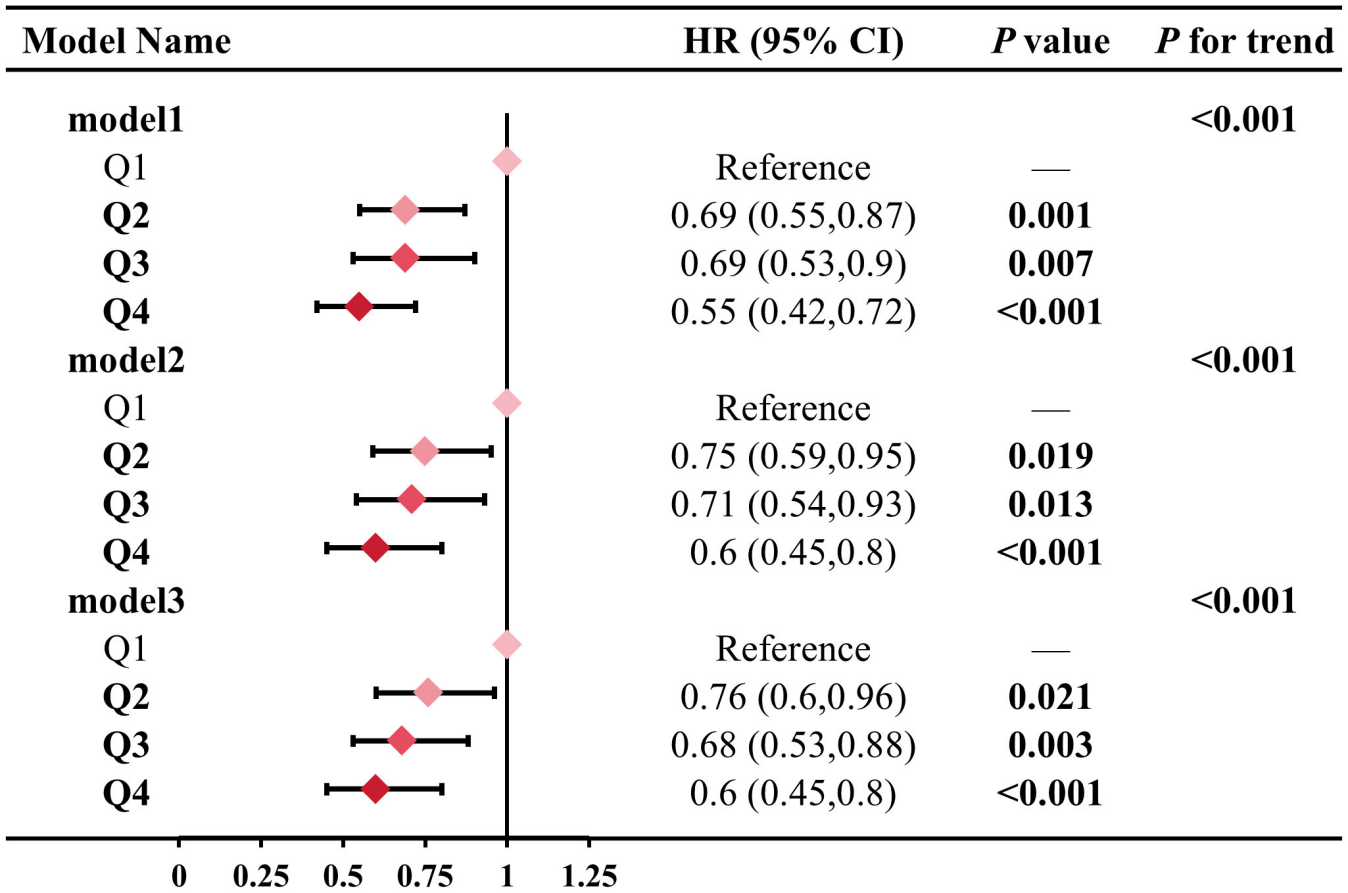
Association between LZ intake levels and all-cause mortality. Model 1 adjusted for age; Model 2 adjusted for model 1, and gender, race, education level, income; Model 3 adjusted for model 2, and smoking, alcohol intake, BMI, total calories. Test for trend based on variable containing median value for each quartile.

Next, we constructed multivariate Cox regression models to assess the relationship between different levels of total LZ intake and all-cause mortality (Figure 5). The results of all three models indicated a significant trend effect for total LZ intake on all-cause mortality (*P* < 0.001). Compared to the Q1 group, the risk of all-cause mortality in the Q4 group was reduced by 0.55 (0.42, 0.72) times, 0.60 (0.45, 0.80) times, and 0.60 (0.45, 0.80) times in the three models, respectively. These results suggest that the level of LZ intake has a significant impact on all-cause mortality risk.

To assess the stability of the results, we performed stratified analyses by different covariates to examine the impact of gender, age, and BMI on the effectiveness of the models (Supplementary Figure S2). For overall biological aging acceleration, gender did not significantly affect the relationship between total LZ intake and biological aging acceleration across the three models (all *P* for interaction > 0.05). However, gender had a significant interaction effect with total LZ intake and all-cause mortality only in Model 1 (*P* for interaction < 0.001). In contrast, different age groups showed significant interaction effects across all models assessing biological aging acceleration (*P* for interaction, Model 1: 0.021; Model 2: 0.016; Model 3: 0.024). The results showed that in the older age group (≥60 years), LZ intake had a more significant impact on biological aging acceleration (*P*, Model 1: 0.002; Model 2: 0.009; Model 3: 0.024), and similar results were observed in the evaluation of the association between different levels of LZ intake and all-cause mortality risk (Age group ≥60 years: all *P* < 0.001). We also noted that in the younger age group (Age group 20–39 years), the relationship between total LZ intake and biological aging acceleration as well as all-cause mortality was not significant. Furthermore, in the evaluation of interaction effects by different BMI categories, no significant effects were found in Model 3 for biological aging acceleration and all-cause mortality.

Additionally, in the evaluation of the effects of different covariates on organ-specific biological aging acceleration (Supplementary Figure S3), we found significant interaction effects of age group on the association between total LZ intake and cardiovascular and kidney biological aging acceleration (*P* for interaction < 0.001). Similarly, in the older age group, total LZ intake had a more significant effect on reducing biological aging acceleration. This result suggests that the benefits of LZ intake are more pronounced in older populations with faster aging progression.

### 3.3 Molecular mechanisms underlying the anti-aging effects of lutein

**Figure 6:**
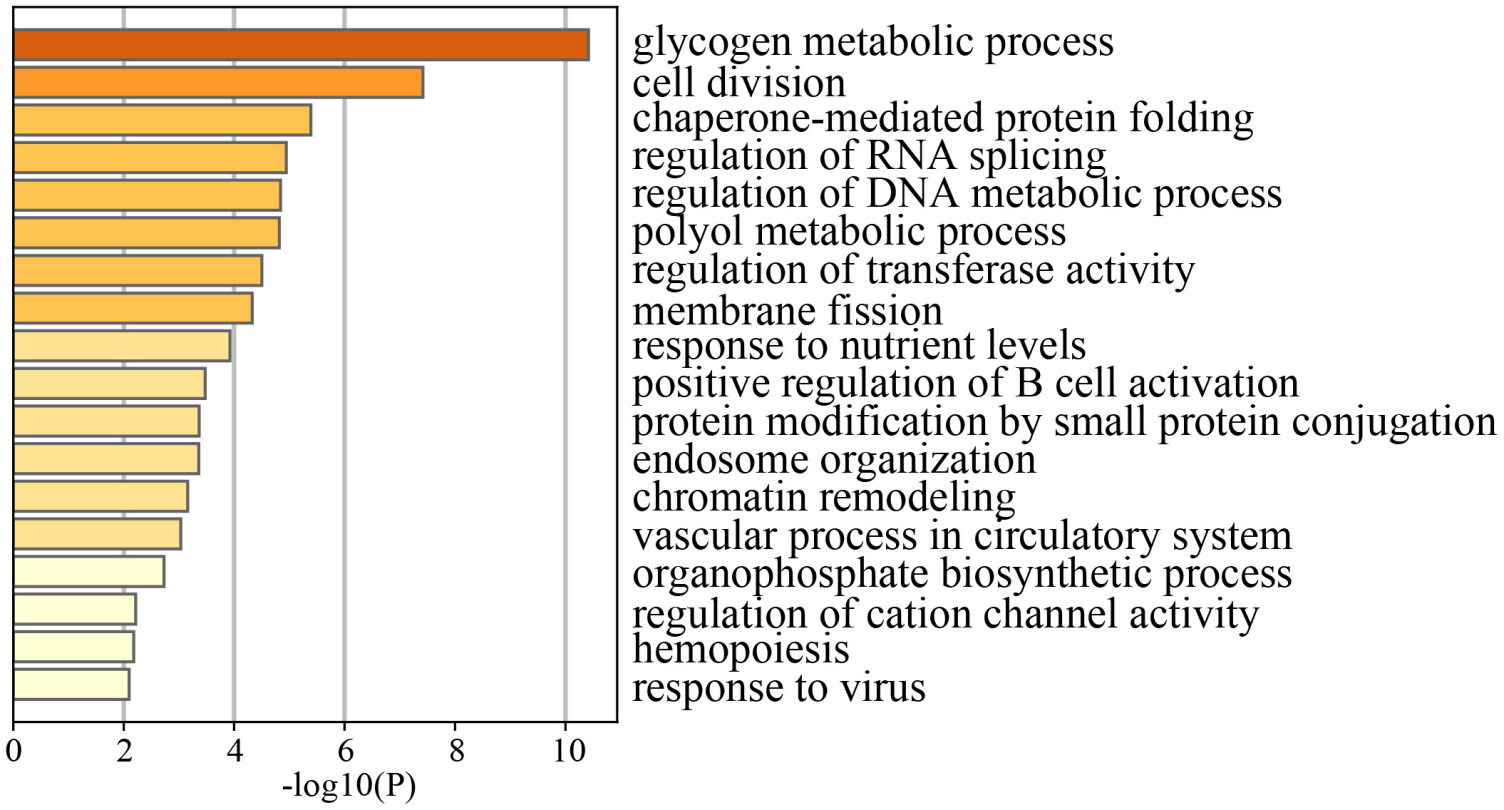
Biological process enrichment analysis at transcriptome level before and after high lutein intake.

To further explore the intrinsic mechanisms by which lutein as main carotenoid mediate the aging process, we collected data from a microarray dataset of genes differentially expressed after 8 weeks of high lutein intake. After performing biological process (BP) enrichment clustering analysis (Figure 6), we found that lutein mainly modulates pathways involved in glycogen metabolic processes, cell division, chaperone-mediated protein folding, regulation of DNA metabolic processes, regulation of transferase activity, chromatin remodeling, and vascular processes in the circulatory system. Further examination of the clustering results (Supplementary Figure S4) revealed that Cluster 5 (regulation of DNA metabolic process) was primarily associated with telomere regulation, suggesting that lutein may help combat aging acceleration by maintaining telomerase activity. GSEA also indicated that lutein intake could upregulate anti-aging related biological processes (Supplementary Figure S5). Furthermore, we observed that aging resistance may also be maintained through certain post-translational modification processes (Supplementary Figure S4).

**Figure 7:**
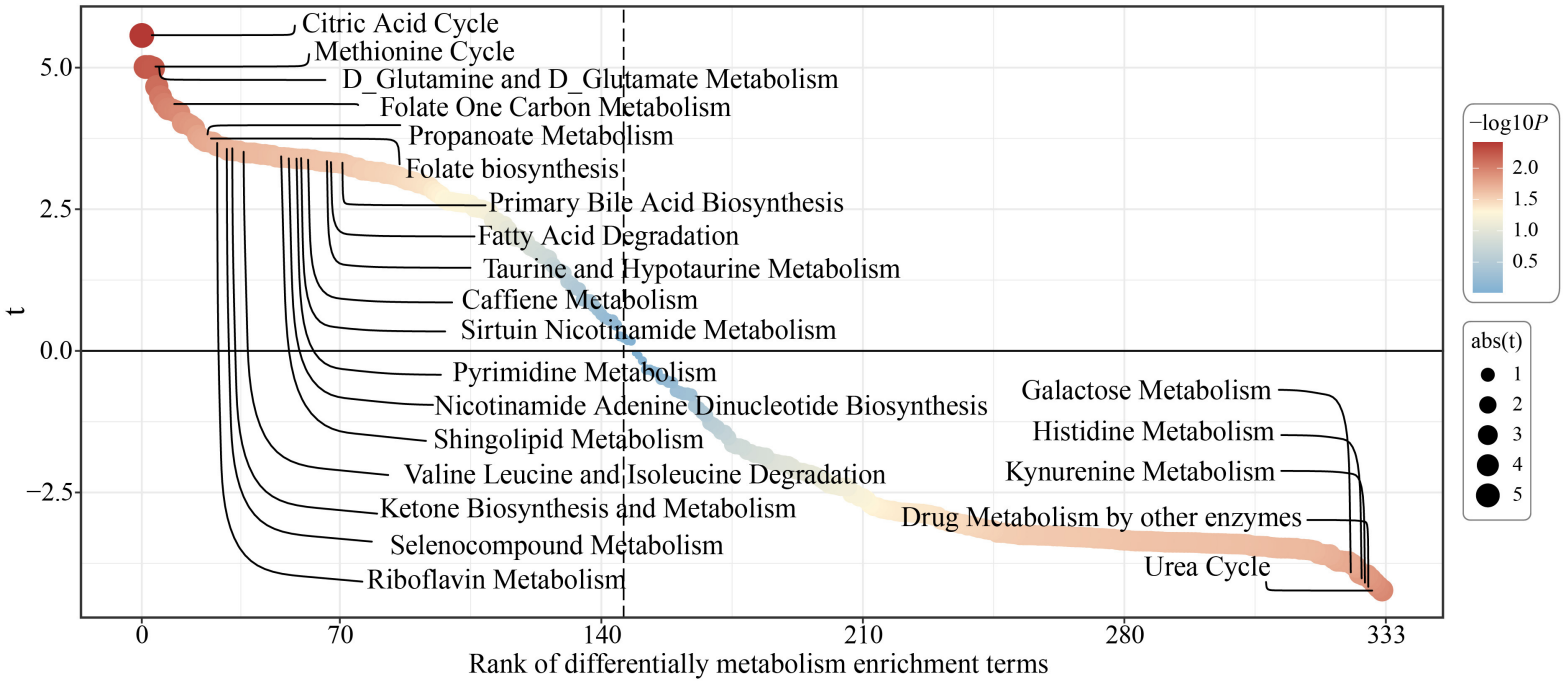
Metabolic pathway enrichment analysis before and after high lutein intake.

The acceleration of the aging process can lead to changes in certain metabolism-related pathways. Therefore, we further investigated the metabolic pathway changes following sustained lutein intake. The results showed (Figure 7) that certain anti-aging metabolic processes were significantly upregulated, such as Folate One Carbon Metabolism, Valine, Leucine, and Isoleucine Degradation, Nicotinamide Adenine Dinucleotide Biosynthesis, Sphingolipid Metabolism, and Primary Bile Acid Biosynthesis. Moreover, the upregulation of fatty acid degradation-related pathways further supports the lipid-lowering effect of lutein (18).

Studies have shown that immune dysregulation can also accelerate the aging process. We continued to explore the impact of sustained lutein intake on immune cell abundance. The results indicated (Supplementary Figure S6) that after 8 weeks of continuous lutein intake, the overall immune score increased compared to the control group. Notably, we observed a downregulation of Th1 cell abundance, as Th1 cells are primarily involved in the body's inflammatory response. This suggests that lutein may, to some extent, delay the aging process through inflammation resistance. The downregulation of the interleukin-mediated inflammatory response pathways further supports this finding (Supplementary Figure S5).

### 3.4 Lutein-mediated changes in aging-related phenotypes

Related studies have shown that the aging process is often accompanied by various changes in phenotypes, including SASP, cell cycle, apoptosis resistance, alterations in cell surface marker expression, and the involvement of certain non-coding RNAs. To investigate this further, we collected various phenotypic markers related to the aging process (22–26) (Supplementary Table S4). The results indicated that after 8 weeks of continuous lutein intake, the expression of most markers in different phenotypes was downregulated (Supplementary Figure S7). The changes in various aging-related phenotypes further confirm the anti-aging efficacy of lutein.

## 4 Discussion

This study explored the relationship between LZ intake and biological aging progression. Using data from the NHANES 2007–2015 database, we quantified biological aging acceleration through the KDM method and further analyzed the potential effects of high lutein intake on aging phenotypes. Our results suggest that lutein intake helps reduce biological aging rates to some extent, with significant anti-aging effects observed in the biological aging processes of the liver, kidney, and cardiovascular organs. Additionally, we found that lutein's role in slowing the biological aging acceleration of organs is mediated through the regulation of multiple molecular mechanisms, including telomere regulation, metabolic pathways, and immune responses.

In this study, we observed that as lutein intake increased, participants' overall biological age acceleration significantly decreased. Specifically, the total intake of LZ was negatively correlated with biological aging acceleration in various organs (such as the cardiovascular system, liver, and kidneys) and overall aging, with this relationship remaining significant after adjusting for multiple covariates. This finding is consistent with previous research, which has shown that carotenoids, particularly lutein, possess antioxidant properties that help delay aging (10, 27). Furthermore, lutein improves endothelial function by lowering blood pressure, thinning arterial walls, and inhibiting vascular smooth muscle cell migration, thus significantly reducing pro-inflammatory cytokine levels (28). We also found that within a certain dose range, lutein intake significantly slows aging, while excessive intake may not provide additional benefits. This suggests the need for optimization in the dosage design of carotenoid interventions.

Molecular mechanism analysis of lutein further elucidates its anti-aging effects. Transcriptomic analysis revealed that sustained lutein intake significantly upregulated biological processes related to anti-aging, including telomere repair, immune regulation, and metabolic homeostasis. For example, lutein intake may combat aging by maintaining telomere stability (29). Additionally, we found that lutein intake helps regulate immune cell abundance, particularly reducing the number of pro-inflammatory Th1 cells, which may slow the aging process by suppressing inflammatory responses. The downregulation of inflammatory factors observed in the GSEA results further supports this finding. Moreover, SASP (senescence-associated secretory phenotype) refers to a set of cytokines and molecules secreted by aging cells, which is closely linked to local and systemic inflammatory responses and the development of several aging-related diseases (30, 31). Regulating SASP formation and clearing senescent cells is a critical strategy to delay aging and prevent age-related diseases. We were excited to find that sustained lutein intake effectively reduced the expression of aging phenotype-related molecules, which further explains the anti-aging effects of lutein.

Despite providing preliminary evidence for the relationship between lutein and biological aging, this study has some limitations. Since it is a cross-sectional study, although we controlled for several confounding factors using PSM and multivariate adjustments, causal relationships still require further validation in future studies. Longitudinal studies will help confirm the causal link between lutein intake and the aging process. Additionally, this cross-sectional study focused only on lutein and zeaxanthin intake, future studies could expand to include other carotenoids to comprehensively assess differences and commonalities in their anti-aging effects. Beyond investigating the anti-aging effects of lutein in human cohort samples, previous studies have also reported that lutein may provide a therapeutic basis for promoting cartilage regeneration and alleviating osteoarthritis in mice. Notably, lutein similarly demonstrates anti-aging properties in aged mice, significantly relieving the aging level of liver, heart, kidney and lung (32).

The inverse association between levels of lutein/zeaxanthin intake and biological aging metrics suggests a protective role of these carotenoids in human aging. It should be noted, however, that translating such observational associations into practical applications—particularly as a dietary supplement—may face regulatory challenges, including demonstrating causal efficacy through randomized controlled trials (RCTs), defining measurable clinical endpoints, and establishing standardized, safe long-term dosing regimens in target populations (33, 34). Despite these translational considerations, our study provides critical epidemiological evidence directly linking lutein intake to attenuated biological aging in humans, thereby establishing a rationale for future interventional studies and mechanistic investigations.

## 5 Conclusion

In conclusion, our study provides novel evidence that combined LZ intake is significantly associated with attenuated multi-level biological aging (OR: 0.93, 95% CI: 0.88–0.98; *P* < 0.05) and reduced all-cause mortality risk (*P* for trend < 0.001). In addition, it implicates that lutein as the primary mediator provide potential benefits in delaying aging through telomere maintenance and inflammation suppression. These findings establish LZ, particularly lutein, as a promising dietary strategy for aging health management, providing reference value for further clinical trials for public health translation.

## Data Availability

The original contributions presented in the study are included in the article/Supplementary material, further inquiries can be directed to the corresponding authors.

## References

[1] Harman D. The aging process: major risk factor for disease and death. Proc Natl Acad Sci U S A. (1991) 88:5360–3. 10.1073/pnas.88.12.5360 2052612 PMC51872

[2] Comfort A. Test-battery to measure ageing-rate in man. Lancet. (1969) 294:1411–5. 10.1016/S0140-6736(69)90950-7 4188291

[3] Cho IH Park KS Lim CJ. An empirical comparative study on biological age estimation algorithms with an application of Work Ability Index (WAI). Mech Ageing Dev. (2010) 131:69–78. 10.1016/j.mad.2009.12.001 20005245

[4] Nakamura E Miyao K. A method for identifying biomarkers of aging and constructing an index of biological age in humans. J Gerontol A Biol Sci Med Sci. (2007) 62:1096–105. 10.1093/gerona/62.10.1096 17921421

[5] Klemera P Doubal S. A new approach to the concept and computation of biological age. Mech Ageing Dev. (2006) 127:240–8. 10.1016/j.mad.2005.10.004 16318865

[6] Bae CY Kang YG Kim S Cho C Kang HC Yu BY. Development of models for predicting biological age (BA) with physical, biochemical, and hormonal parameters. Arch Gerontol Geriatr. (2008) 47:253–65. 10.1016/j.archger.2007.08.009 17889950

[7] Levine ME. Modeling the rate of senescence: can estimated biological age predict mortality more accurately than chronological age? J Gerontol A Biol Sci Med Sci. (2013) 68:667–74. 10.1093/gerona/gls233 23213031 PMC3660119

[8] Song L Zhang S. Anti-aging activity and modes of action of compounds from natural food sources. Biomolecules. (2023) 13:1600. 10.3390/biom13111600 38002283 PMC10669485

[9] Ames BN. Prolonging healthy aging: longevity vitamins and proteins. Proc Natl Acad Sci U S A. (2018) 115:10836–44. 10.1073/pnas.1809045115 30322941 PMC6205492

[10] Thomas SE Johnson EJ. Xanthophylls. Adv Nutr. (2018) 9:160–2. 10.1093/advances/nmx005 29659682 PMC5916423

[11] Fuad NIN Sekar M Gan SH Lum PT Vaijanathappa J Ravi S. Lutein: a comprehensive review on its chemical, biological activities and therapeutic potentials. Pharmacogn J. (2020). Available online at: https://ir.unikl.edu.my/xmlui/handle/123456789/25184

[12] Schwartz S Frank E Gierhart D Simpson P Frumento R. Zeaxanthin-based dietary supplement and topical serum improve hydration and reduce wrinkle count in female subjects. J Cosmet Dermatol. (2016) 15:e13–20. 10.1111/jocd.12226 27312122

[13] Ralston SH Corral-Gudino L Cooper C Francis RM Fraser WD Gennari L. Diagnosis and management of paget's disease of bone in adults: a clinical guideline. J Bone Miner Res. (2019) 34:579–604. 10.1002/jbmr.3657 30803025 PMC6522384

[14] Lowe D Sanvictores T Zubair M John S. Alkaline Phosphatase. Treasure Island, FL: StatPearls Publishing LLC. (2025). 29083622

[15] Nie C Li Y Li R Yan Y Zhang D Li T. Distinct biological ages of organs and systems identified from a multi-omics study. Cell Rep. (2022) 38:110459. 10.1016/j.celrep.2022.110459 35263580

[16] Soda R Tavassoli M. Liver endothelium and not hepatocytes or Kupffer cells have transferrin receptors. Blood. (1984) 63:270–6. 10.1182/blood.V63.2.270.270 6318865

[17] Davis S Meltzer PS. GEOquery: a bridge between the Gene Expression Omnibus (GEO) and BioConductor. Bioinformatics. (2007) 23:1846–7. 10.1093/bioinformatics/btm254 17496320

[18] Takagi T Hayashi R Nakai Y Okada S Miyashita R Yamada M. Dietary intake of carotenoid-rich vegetables reduces visceral adiposity in obese Japanese men-a randomized, double-blind trial. Nutrients. (2020) 12:2342. 10.3390/nu12082342 32764462 PMC7468729

[19] Ritchie ME Phipson B Wu D Hu Y Law CW Shi W. limma powers differential expression analyses for RNA-sequencing and microarray studies. Nucleic Acids Res. (2015) 43:e47. 10.1093/nar/gkv007 25605792 PMC4402510

[20] Yu G Wang LG Han Y He QY. clusterProfiler: an R package for comparing biological themes among gene clusters. OMICS. (2012) 16:284–7. 10.1089/omi.2011.0118 22455463 PMC3339379

[21] Zeng D Ye Z Shen R Yu G Wu J Xiong Y. IOBR: multi-omics immuno-oncology biological research to decode tumor microenvironment and signatures. Front Immunol. (2021) 12:687975. 10.3389/fimmu.2021.687975 34276676 PMC8283787

[22] Guan L Crasta KC Maier AB. Assessment of cell cycle regulators in human peripheral blood cells as markers of cellular senescence. Ageing Res Rev. (2022) 78:101634. 10.1016/j.arr.2022.101634 35460888

[23] Hernandez-Segura A de Jong TV Melov S Guryev V Campisi J Demaria M. Unmasking transcriptional heterogeneity in senescent cells. Curr Boil. (2017) 27:2652-60.e4. 10.1016/j.cub.2017.07.033 28844647 PMC5788810

[24] Casella G Munk R Kim KM Piao Y De S Abdelmohsen K. Transcriptome signature of cellular senescence. Nucleic Acids Res. (2019) 47:7294–305. 10.1093/nar/gkz555 31251810 PMC6698740

[25] Gorgoulis V Adams PD Alimonti A Bennett DC Bischof O Bishop C. cellular senescence: defining a path forward. Cell. (2019) 179:813–27. 10.1016/j.cell.2019.10.005 31675495

[26] Suryadevara V Hudgins AD Rajesh A Pappalardo A Karpova A Dey AK. SenNet recommendations for detecting senescent cells in different tissues. Nat Rev Mol Cell Biol. (2024) 25:1001–23. 10.1038/s41580-024-00738-8 38831121 PMC11578798

[27] Bakac ER Percin E Gunes-Bayir A Dadak A. A narrative review: the effect and importance of carotenoids on aging and aging-related diseases. Int J Mol Sci. (2023) 24:15199. 10.3390/ijms242015199 37894880 PMC10607816

[28] Hajizadeh-Sharafabad F Ghoreishi Z Maleki V Tarighat-Esfanjani A. Mechanistic insights into the effect of lutein on atherosclerosis, vascular dysfunction, and related risk factors: a systematic review of *in vivo, ex vivo* and *in vitro* studies. Pharmacol Res. (2019) 149:104477. 10.1016/j.phrs.2019.104477 31605782

[29] Sen A Marsche G Freudenberger P Schallert M Toeglhofer AM Nagl C. Association between higher plasma lutein, zeaxanthin, and vitamin C concentrations and longer telomere length: results of the Austrian Stroke Prevention Study. J Am Geriatr Soc. (2014) 62:222–9. 10.1111/jgs.12644 24428184 PMC4234001

[30] Coppé JP Desprez PY Krtolica A Campisi J. The senescence-associated secretory phenotype: the dark side of tumor suppression. Annu Rev Pathol. (2010) 5:99–118. 10.1146/annurev-pathol-121808-102144 20078217 PMC4166495

[31] Kuilman T Peeper DS. Senescence-messaging secretome: SMS-ing cellular stress. Nat Rev Cancer. (2009) 9:81–94. 10.1038/nrc2560 19132009

[32] Zhao K Zhou T Yang J Li Y Qin J Wang S.. Lutein shows a protective effect against the aging of mesenchymal stem cells by downregulating inflammation. Int Immunopharmacol. (2023) 116:109749. 10.1016/j.intimp.2023.109749

[33] Durazzo A Sorkin BC Lucarini M Gusev PA Kuszak AJ Crawford C. Analytical challenges and metrological approaches to ensuring dietary supplement quality: international perspectives. Front Pharmacol. (2021) 12:714434. 10.3389/fphar.2021.714434 35087401 PMC8787362

[34] Dwyer JT Coates PM Smith MJ. Dietary supplements: regulatory challenges and research resources. Nutrients. (2018) 10:41. 10.3390/nu10010041 29300341 PMC5793269

