# Supplementary material for: The association between lutein and zeaxanthin intake and multi-level biological aging
Source: Front Nutr. 2025 Aug 8;12:1618158. doi: 10.3389/fnut.2025.1618158 (PMC12370510; doi:10.3389/fnut.2025.1618158)
Supplement: Supplementary file 1 [file Table_1.docx]

Supplementary Material

**The Association Between Lutein and Zeaxanthin Intake and Multi-level Biological Aging**

## Supplementary Tables

**Supplementary Table 1.** Baseline characteristics of participants by LZ total intake.

| **Characteristic** | **Q1** N = 20,325,492 (25%)^1^ | **Q2** N = 20,301,191 (25%)^1^ | **Q3** N = 20,313,592 (25%)^1^ | **Q4** N = 20,298,178 (25%)^1^ | ***P value****^2^* | |
| --- | --- | --- | --- | --- | --- | --- |
| **Age.group** |  |  |  |  | **<0.001** | |
| 20-39 years | 8,666,889 (43%) | 7,376,195 (36%) | 5,946,082 (29%) | 5,749,481 (28%) |  | |
| 40-59 years | 7,432,170 (37%) | 7,375,184 (36%) | 7,950,227 (39%) | 8,146,119 (40%) |  | |
| ≥60 years | 4,226,433 (21%) | 5,549,811 (27%) | 6,417,283 (32%) | 6,402,578 (32%) |  | |
| **Sex** |  |  |  |  | **<0.001** | |
| female | 10,596,690 (52%) | 10,624,239 (52%) | 9,330,920 (46%) | 11,330,272 (56%) |  | |
| male | 9,728,801 (48%) | 9,676,952 (48%) | 10,982,672 (54%) | 8,967,906 (44%) |  | |
| **Race** |  |  |  |  | **<0.001** | |
| Non-Hispanic White | 13,794,695 (68%) | 14,140,572 (70%) | 14,334,158 (71%) | 14,973,161 (74%) |  | |
| Non-Hispanic Black | 2,550,706 (13%) | 1,932,380 (9.5%) | 1,726,727 (8.5%) | 1,757,522 (8.7%) |  | |
| Mexican American | 1,687,652 (8.3%) | 1,880,009 (9.3%) | 1,671,872 (8.2%) | 961,465 (4.7%) |  | |
| Other Hispanic | 1,318,208 (6.5%) | 1,242,647 (6.1%) | 984,146 (4.8%) | 786,798 (3.9%) |  | |
| Other/multiracial | 974,231 (4.8%) | 1,105,584 (5.4%) | 1,596,689 (7.9%) | 1,819,233 (9.0%) |  | |
| **Education.attainment** |  |  |  |  | **<0.001** | |
| Less Than 9th Grade | 1,462,666 (7.2%) | 1,046,300 (5.2%) | 892,447 (4.4%) | 510,506 (2.5%) |  | |
| 9-11th Grade | 3,070,825 (15%) | 2,638,558 (13%) | 2,085,451 (10%) | 1,182,368 (5.8%) |  | |
| High School Grad/GED | 5,695,009 (28%) | 4,702,467 (23%) | 4,554,650 (22%) | 3,040,929 (15%) |  | |
| Some College or  AA degree | 6,398,955 (31%) | 6,536,608 (32%) | 6,627,868 (33%) | 5,786,407 (29%) |  | |
| College Graduate or above | 3,698,037 (18%) | 5,377,258 (26%) | 6,153,175 (30%) | 9,777,969 (48%) |  | |
| **BMI.group** |  |  |  |  | **0.004** | |
| Underweight(<18.5) | 431,115 (2.1%) | 276,387 (1.4%) | 285,186 (1.4%) | 410,904 (2.0%) |  | |
| Normal(18.5 to <25) | 5,412,858 (27%) | 5,281,424 (26%) | 5,772,186 (28%) | 6,749,359 (33%) |  | |
| Overweight(25 to <30) | 6,735,749 (33%) | 6,752,071 (33%) | 6,936,674 (34%) | 6,531,851 (32%) |  | |
| Obese(30 or greater) | 7,745,770 (38%) | 7,991,309 (39%) | 7,319,546 (36%) | 6,606,064 (33%) |  | |
| **Alq.group** |  |  |  |  | **<0.001** | |
| Non-drinker | 4,873,389 (24%) | 4,452,171 (22%) | 4,504,943 (22%) | 4,258,638 (21%) |  | |
| <1 drinks/month | 5,285,128 (26%) | 4,697,190 (23%) | 4,290,501 (21%) | 3,219,116 (16%) |  | |
| 1-10 drinks/month | 7,425,819 (37%) | 7,870,382 (39%) | 7,454,412 (37%) | 8,183,215 (40%) |  | |
| >10 drinks/month | 2,741,155 (13%) | 3,281,448 (16%) | 4,063,737 (20%) | 4,637,209 (23%) |  | |
| **Smoke.group** |  |  |  |  | **<0.001** | |
| Current smoker | 7,559,864 (37%) | 6,336,628 (31%) | 5,250,922 (26%) | 4,331,695 (21%) |  | |
| Former smoker | 2,515,920 (12%) | 3,266,293 (16%) | 4,116,323 (20%) | 4,086,712 (20%) |  | |
| Never smoker | 10,249,708 (50%) | 10,698,270 (53%) | 10,946,347 (54%) | 11,879,772 (59%) |  | |
| **Age** | 44 (29, 56) | 47 (33, 61) | 51 (37, 64) | 52 (38, 63) | **<0.001** | |
| **PIR** | 2.15 (1.07, 4.10) | 2.72 (1.32, 4.72) | 3.14 (1.63, 5.00) | 3.93 (2.04, 5.00) | **<0.001** | |
| **BMI** | 28 (24, 33) | 28 (25, 33) | 28 (24, 32) | 27 (24, 32) | **0.001** | |
| **Waist** | 99 (87, 110) | 99 (89, 109) | 98 (88, 109) | 96 (86, 107) | **0.003** | |
| **Total.calories** | 1,724 (1,315, 2,244) | 1,994 (1,554, 2,499) | 2,104 (1,641, 2,704) | 2,019 (1,603, 2,598) | **<0.001** | |
| **Dietary.LZ** | 0.30 (0.18, 0.38) | 0.65 (0.55, 0.76) | 1.18 (0.99, 1.42) | 2.95 (2.14, 4.89) | **<0.001** | |
| **Supplement.LZ** | 0.25 (0.25, 0.25) | 0.25 (0.25, 0.25) | 0.25 (0.25, 0.30) | 0.30 (0.25, 2.00) | **<0.001** | |
| **Total.LZ** | 0.30 (0.19, 0.39) | 0.67 (0.57, 0.78) | 1.23 (1.04, 1.46) | 3.27 (2.33, 5.68) | **<0.001** |  |
| ^1^n (%); Median (Q1, Q3) | | | | | |  |
| ^2^Pearson's X^2: Rao & Scott adjustment; Design-based KruskalWallis test | | | | | |  |

**Supplementary Table 2.** Characteristics of biological age acceleration under different L/Z dietary intake levels before and after the treatment of properness score matching.

| **Dietary L/Z Intake** | **Q1**^1^ | **Q2**^1^ | **Q3**^1^ | **Q4**^1^ | ***P* value**^2^ |
| --- | --- | --- | --- | --- | --- |
| **Before propensity score matching** | | | | | |
| Dietary.LZ | 0.29 (0.18, 0.37) | 0.64 (0.54, 0.73) | 1.14 (0.97, 1.36) | 2.95 (2.14, 4.89) | **<0.001** |
| BA_ratio.Cardio | 0.99 (0.82, 1.20) | 0.99 (0.81, 1.20) | 0.96 (0.79, 1.17) | 0.96 (0.80, 1.13) | **0.025** |
| BA_ratio.Kidney | 1.00 (0.88, 1.17) | 0.99 (0.88, 1.13) | 0.98 (0.88, 1.12) | 0.98 (0.86, 1.11) | **0.002** |
| BA_ratio.Liver | 0.90 (0.55, 1.21) | 0.85 (0.55, 1.19) | 0.84 (0.58, 1.14) | 0.80 (0.54, 1.08) | **<0.001** |
| BA_ratio.Total | 1.00 (0.84, 1.17) | 0.98 (0.84, 1.14) | 0.96 (0.82, 1.13) | 0.94 (0.81, 1.11) | **0.001** |
| **After propensity score matching** | | | | | |
| Dietary.LZ | 0.31 (0.20, 0.39) | 0.65 (0.55, 0.74) | 1.12 (0.97, 1.32) | 2.83 (2.06, 4.56) | **<0.001** |
| BA_ratio.Cardio | 0.99 (0.83, 1.19) | 0.98 (0.81, 1.17) | 0.98 (0.80, 1.19) | 0.97 (0.81, 1.14) | 0.2 |
| BA_ratio.Kidney | 1.00 (0.89, 1.16) | 1.00 (0.88, 1.13) | 0.98 (0.88, 1.13) | 0.98 (0.86, 1.13) | 0.1 |
| BA_ratio.Liver | 0.89 (0.56, 1.18) | 0.85 (0.56, 1.17) | 0.84 (0.57, 1.15) | 0.82 (0.54, 1.14) | 0.2 |
| BA_ratio.Total | 0.99 (0.84, 1.16) | 0.98 (0.84, 1.13) | 0.97 (0.82, 1.14) | 0.95 (0.82, 1.13) | **0.025** |
| ^1^Median (Q1, Q3) | | | | | |
| ^2^Design-based KruskalWallis test | | | | | |

**Supplementary Table 3.** Association between L/Z total intake levels and the biological age acceleration of different organs.

| **Model Name** | **OR (95% CI)** | ***P* value** | ***P* for trend**^1^ |
| --- | --- | --- | --- |
| **BA acceleration.Cardio** | | | |
| **Model 1** |  |  | 0.2 |
| Q1 | Reference | — |  |
| Q2 | 0.77 (0.19,3.07) | 0.7 |  |
| Q3 | 0.81 (0.19,3.44) | 0.8 |  |
| Q4 | 0.37 (0.09,1.53) | 0.2 |  |
| **Model 2** |  |  | 0.2 |
| Q1 | Reference | — |  |
| Q2 | 1.03 (0.25,4.23) | >0.9 |  |
| Q3 | 0.69 (0.17,2.85) | 0.6 |  |
| Q4 | 0.57 (0.13,2.49) | 0.4 |  |
| **Model 3** |  |  | 0.4 |
| Q1 | Reference | — |  |
| Q2 | 0.78 (0.19,3.1) | 0.7 |  |
| Q3 | 0.65 (0.17,2.51) | 0.5 |  |
| Q4 | 0.57 (0.13,2.41) | 0.4 |  |
| **BA acceleration.Kidney** | | | |
| **Model 1** |  |  | **<0.001** |
| Q1 | Reference | — |  |
| Q2 | 0.51 (0.21,1.26) | 0.14 |  |
| **Q3** | 0.28 (0.12,0.65) | **0.004** |  |
| **Q4** | 0.18 (0.07,0.45) | **<0.001** |  |
| **Model 2** |  |  | **<0.001** |
| Q1 | Reference | — |  |
| Q2 | 0.6 (0.25,1.45) | 0.2 |  |
| **Q3** | 0.3 (0.13,0.69) | **0.006** |  |
| **Q4** | 0.19 (0.08,0.46) | **<0.001** |  |
| **Model 3** |  |  | **0.005** |
| Q1 | Reference | — |  |
| Q2 | 0.68 (0.28,1.65) | 0.4 |  |
| Q3 | 0.49 (0.2,1.19) | 0.11 |  |
| **Q4** | 0.3 (0.12,0.7) | **0.007** |  |
| **BA acceleration.Liver** | | | |
| **Model 1** |  |  | **0.016** |
| Q1 | Reference | — |  |
| Q2 | 0.08 (0.01,1.16) | 0.064 |  |
| Q3 | 0.43 (0.02,10.2) | 0.6 |  |
| **Q4** | 0.02 (0,0.26) | **0.003** |  |
| **Model 2** |  |  | **0.035** |
| Q1 | Reference | — |  |
| Q2 | 0.15 (0.01,2.27) | 0.2 |  |
| Q3 | 0.42 (0.02,9.56) | 0.6 |  |
| **Q4** | 0.04 (0,0.52) | **0.014** |  |
| **Model 3** |  |  | **0.041** |
| Q1 | Reference | — |  |
| **Q2** | 0.07 (0.01,0.98) | **0.048** |  |
| Q3 | 0.35 (0.01,9.3) | 0.5 |  |
| **Q4** | 0.05 (0,0.44) | **0.008** |  |

^1^Test for trend based on variable containing median value for each quartile.

Model 1 adjusted for age;

Model 2 adjusted for model 1, and gender, race, education level, income;

Model 3 adjusted for model 2, and smoking, alcohol intake, BMI, total calories.

**Supplementary Table 4.** Aging-related marker before and after high lutein intake.

| **Gene_Symbol** | **log2FC** | ***P* value** |  | **group** |
| --- | --- | --- | --- | --- |
| **CDKN1A** | **-0.313** | **<0.05** |  | **Cell cycle** |
| **CDKN2A** | **-0.040** | **<0.05** |  |  |
| **TP53** | **-0.032** | **<0.05** |  |  |
| **MKI67** | **-0.029** | **<0.05** |  |  |
| **RB1** | **0.404** | **<0.05** |  |  |
| **IL11** | **-0.033** | **<0.05** |  | **Interleukins (SASP)** |
| **IL6** | **-0.032** | **<0.05** |  |  |
| **IL13** | **-0.026** | **<0.05** |  |  |
| **IL1A** | **-0.018** | **<0.05** |  |  |
| **IL15** | **0.332** | **<0.05** |  |  |
| **IL7** | >-0.01 | ns |  |  |
| **IL1B** | 0.068 | ns |  |  |
| **CCL13** | **-0.029** | **<0.05** |  | **Chemokines (SASP)** |
| **CCL26** | **-0.023** | **<0.05** |  |  |
| **CCL25** | **-0.019** | **<0.05** |  |  |
| **CCL1** | **-0.018** | **<0.05** |  |  |
| **CXCL3** | **-0.016** | **<0.05** |  |  |
| **CCL3** | -0.029 | ns |  |  |
| **CXCL1** | -0.016 | ns |  |  |
| **CCL20** | >-0.01 | ns |  |  |
| **CCL16** | >-0.01 | ns |  |  |
| **CXCL2** | >-0.01 | ns |  |  |
| **CCL8** | >-0.01 | ns |  |  |
| **CCL11** | >-0.01 | ns |  |  |
| **CXCL11** | >-0.01 | ns |  |  |
| **CXCL5** | 0.041 | ns |  |  |
| **CXCL8** | 0.349 | ns |  |  |
| **TGFB1** | **-0.360** | **<0.05** |  | **Other inflammatory molecules (SASP)** |
| **CSF3** | **-0.046** | **<0.05** |  |  |
| **CSF2** | **-0.040** | **<0.05** |  |  |
| **IFNG** | **-0.014** | **<0.05** |  |  |
| **GDF15** | **-0.014** | **<0.05** |  |  |
| **MIF** | -0.170 | ns |  |  |
| **CXCL13** | >-0.01 | ns |  |  |
| **IGFBP3** | **-0.048** | **<0.05** |  | **Growth factors; regulators (SASP)** |
| **IGFBP4** | **-0.044** | **<0.05** |  |  |
| **NGF** | **-0.043** | **<0.05** |  |  |
| **IGFBP6** | **-0.030** | **<0.05** |  |  |
| **IGFBP2** | **-0.022** | **<0.05** |  |  |
| **EREG** | **-0.021** | **<0.05** |  |  |
| **ANG** | **-0.021** | **<0.05** |  |  |
| **FGF2** | **-0.013** | **<0.05** |  |  |
| **CXCL12** | **-0.012** | **<0.05** |  |  |
| **PIGF** | **0.230** | **<0.05** |  |  |
| **IGFBP7** | -0.040 | ns |  |  |
| **NRG1** | -0.019 | ns |  |  |
| **EGF** | >-0.01 | ns |  |  |
| **KITLG** | >-0.01 | ns |  |  |
| **AREG** | >-0.01 | ns |  |  |
| **FGF7** | >-0.01 | ns |  |  |
| **HGF** | 0.010 | ns |  |  |
| **VEGFA** | 0.024 | ns |  |  |
| **TIMP1** | **-0.506** | **<0.05** |  | **Proteases and regulators (SASP)** |
| **TIMP2** | **-0.372** | **<0.05** |  |  |
| **MMP14** | **-0.075** | **<0.05** |  |  |
| **SERPINB2** | **-0.038** | **<0.05** |  |  |
| **PLAU** | **-0.021** | **<0.05** |  |  |
| **SERPINE1** | **-0.013** | **<0.05** |  |  |
| **MMP13** | **>-0.01** | **<0.05** |  |  |
| **CTSB** | -0.451 | ns |  |  |
| **MMP10** | >-0.01 | ns |  |  |
| **PLAT** | >-0.01 | ns |  |  |
| **MMP12** | >-0.01 | ns |  |  |
| **MMP3** | >-0.01 | ns |  |  |
| **MMP1** | >-0.01 | ns |  |  |
| **TNFRSF1B** | **-0.464** | **<0.05** |  | **Receptors; ligands (SASP)** |
| **ICAM3** | **-0.365** | **<0.05** |  |  |
| **TNFRSF10C** | **-0.252** | **<0.05** |  |  |
| **PLAUR** | **-0.244** | **<0.05** |  |  |
| **TNFRSF1A** | **-0.231** | **<0.05** |  |  |
| **ICAM1** | **-0.080** | **<0.05** |  |  |
| **EGFR** | **-0.020** | **<0.05** |  |  |
| **IL6ST** | **0.141** | **<0.05** |  |  |
| **FAS** | **0.752** | **<0.05** |  |  |
| **TNFRSF11B** | >-0.01 | ns |  |  |
| **PIK3CD** | **-0.157** | **<0.05** |  | **Apoptosis-related molecules** |
| **ABL1** | **-0.143** | **<0.05** |  |  |
| **EFNB3** | **-0.041** | **<0.05** |  |  |
| **EFNB1** | **-0.022** | **<0.05** |  |  |
| **BCL2L1** | -0.128 | ns |  |  |
| **BCL2L2** | -0.101 | ns |  |  |
| **BCL2** | <0.01 | ns |  |  |
| **SLC9A3R1** | **-0.195** | ***P* <0.05** |  | **Cell surface markers** |
| **NOTCH1** | **-0.165** | ***P* <0.05** |  |  |
| **LAT2** | **-0.121** | ***P* <0.05** |  |  |
| **PTPRJ** | **-0.115** | ***P* <0.05** |  |  |
| **STX4** | **-0.105** | ***P* <0.05** |  |  |
| **MICA** | **-0.101** | ***P* <0.05** |  |  |
| **NOTCH3** | **-0.063** | ***P* <0.05** |  |  |
| **ULBP2** | **-0.031** | ***P* <0.05** |  |  |
| **TNFRSF10D** | **-0.023** | ***P* <0.05** |  |  |
| **PDCD1LG2** | **-0.019** | ***P* <0.05** |  |  |
| **ARMCX3** | **0.264** | ***P* <0.05** |  |  |
| **DPP4** | -0.026 | ns |  |  |
| **CD274** | 0.065 | ns |  |  |
| **CD36** | 0.090 | ns |  |  |
| **VAMP3** | 0.466 | ns |  |  |
| **MIR424** | **-0.040** | ***P* <0.05** |  | **microRNA** |
| **MIR146A** | **-0.037** | ***P* <0.05** |  |  |
| **MIR34A** | **-0.025** | ***P* <0.05** |  |  |
| **UCA1** | **-0.024** | ***P* <0.05** |  | **lncRNA** |
| **CDKN2B-AS1** | >-0.01 | ns |  |  |

## Supplementary Figures


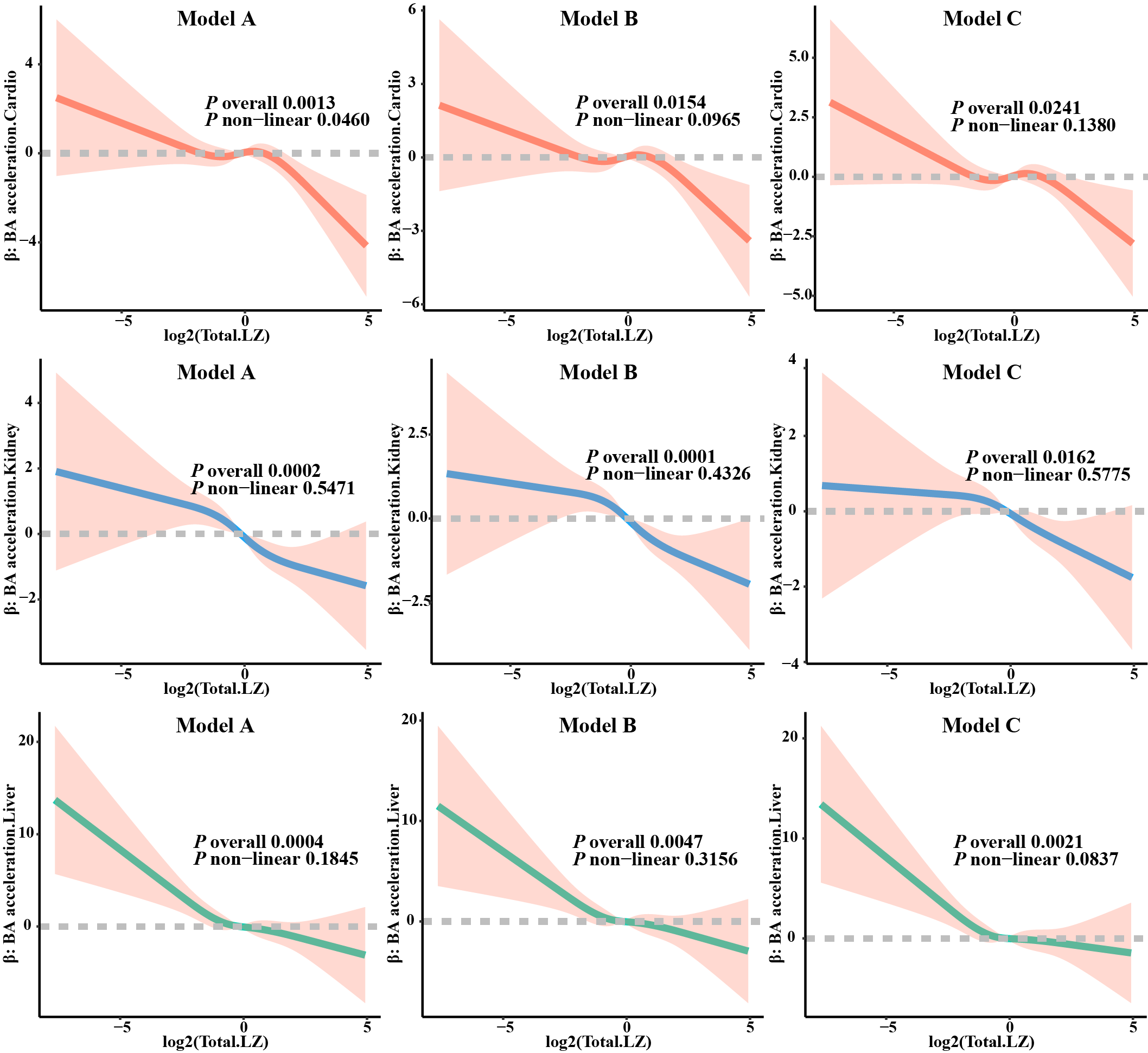


**Supplementary Figure 1.** Analysis of restricted cubic spline regression between LZ total intake levels and organ biological age acceleration. Model 1 adjusted for age; Model 2 adjusted for model 1, and gender, race, education level, income; Model 3 adjusted for model 2, and smoking, alcohol intake, BMI, total calories.


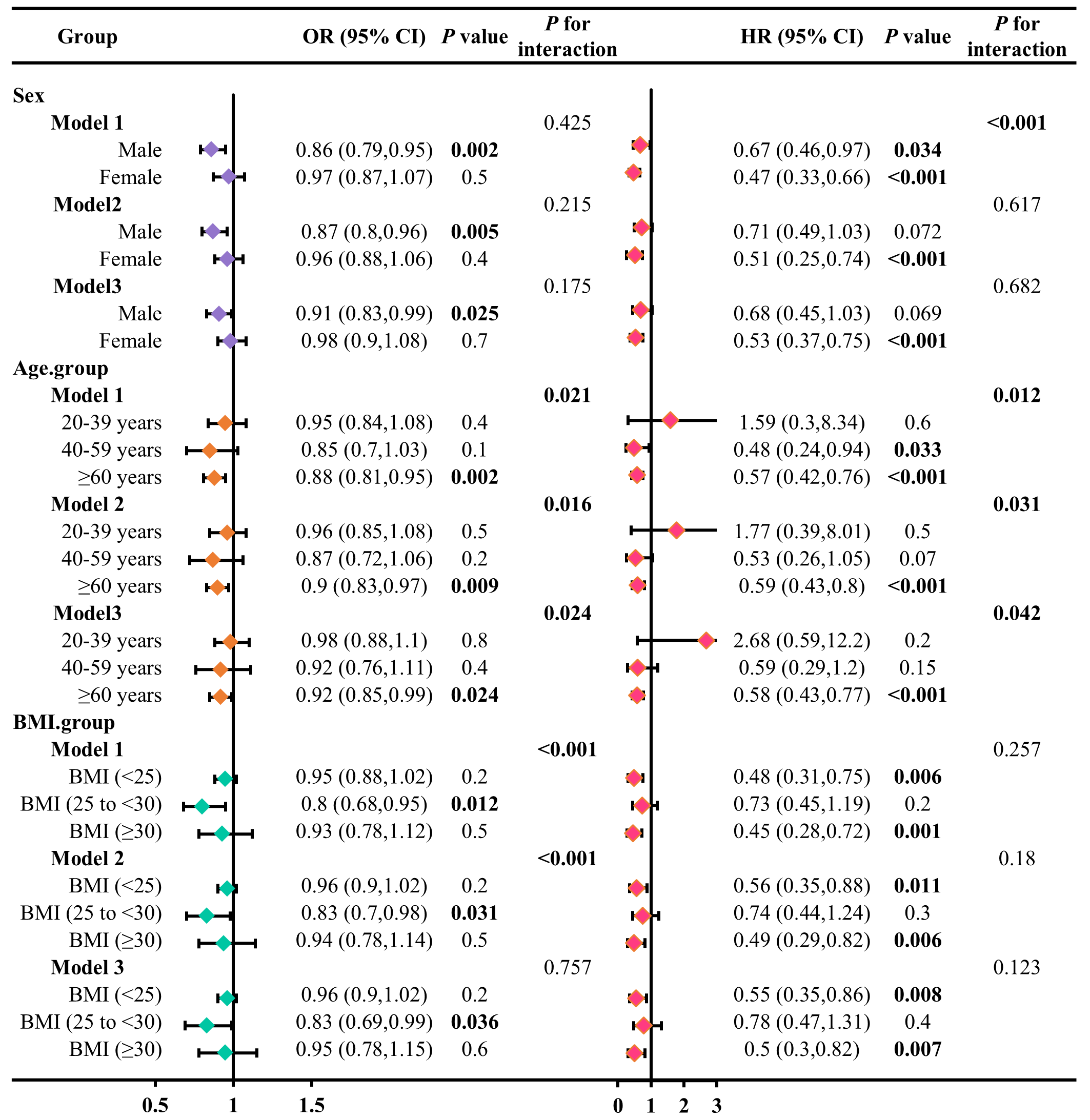


**Supplementary Figure 2**. Association between total L/Z intake levels and overall biological age acceleration, as well as all-cause mortality, stratified by different covariates (sex, age, BMI). The results for all-cause mortality are based on the assessment of L/Z intake between Q4 and Q1. Additionally, the fully adjusted model was used, which included age, sex, race, education, income, smoking, alcohol intake, BMI, and total calories as covariates.


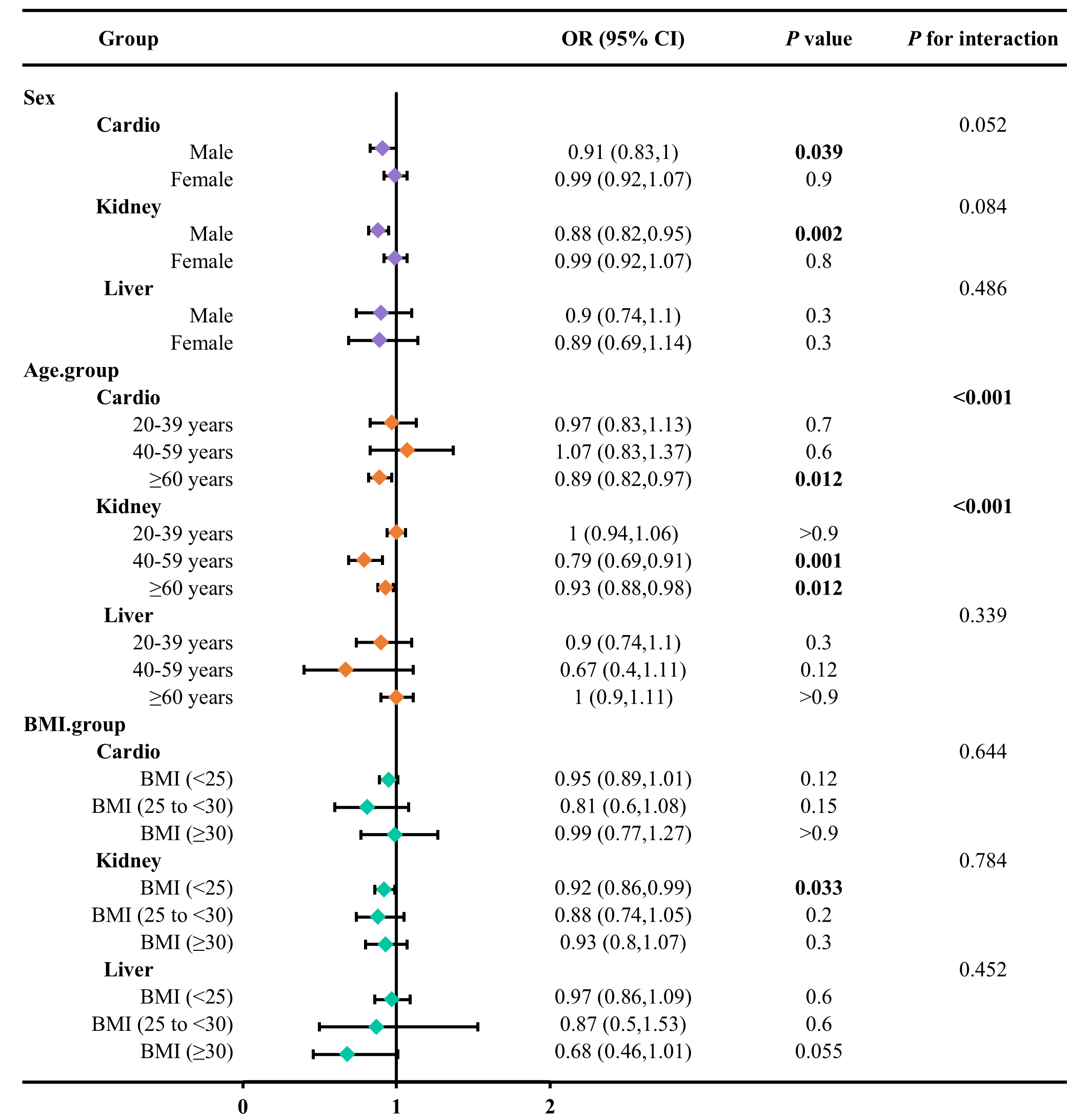


**Supplementary Figure 3.** Association between total L/Z intake levels and the biological age acceleration of different organs, stratified by different covariates (sex, age, BMI). The fully adjusted model was used, which included age, sex, race, education, income, smoking, alcohol intake, BMI, and total calories as covariates.


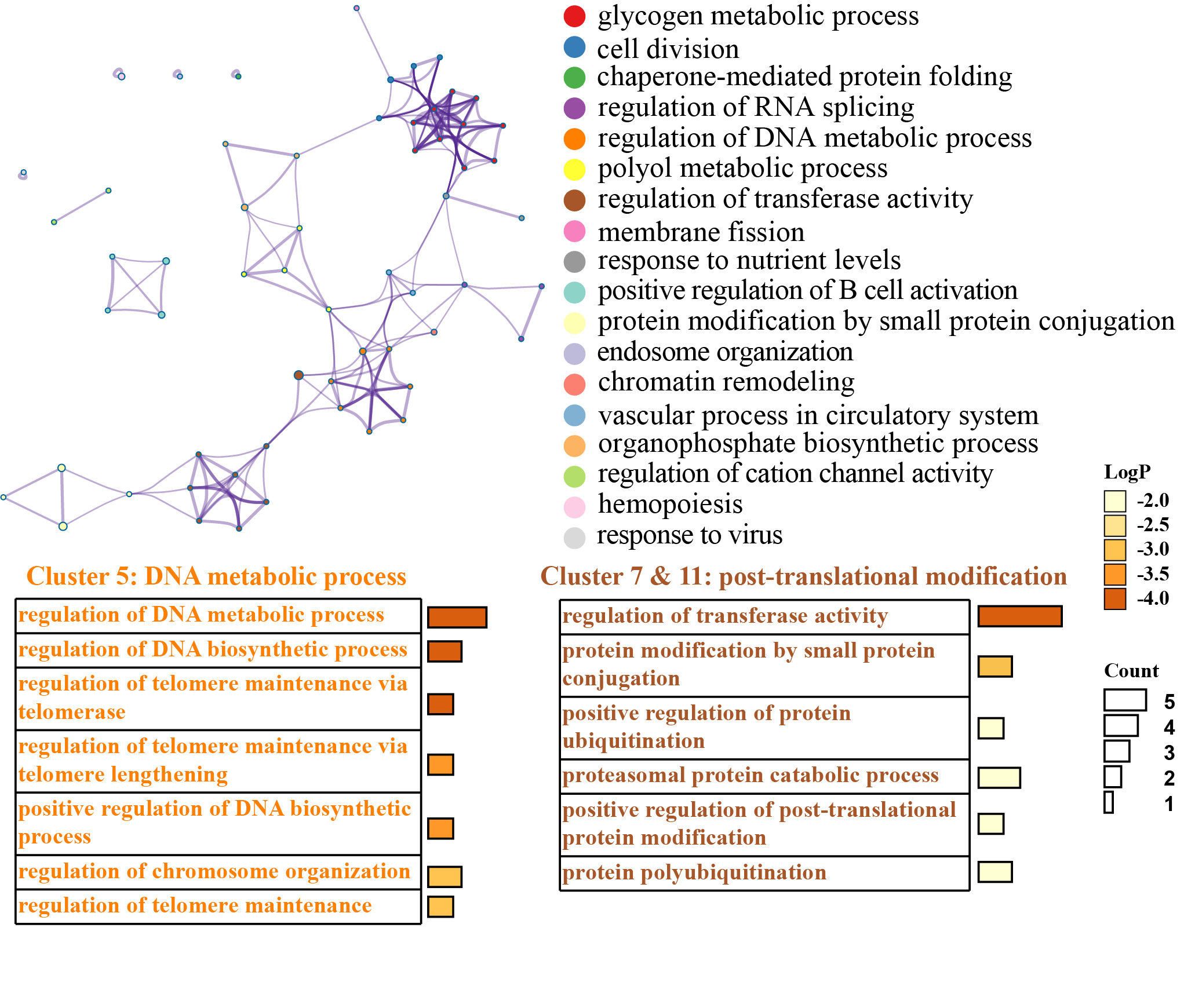


**Supplementary Figure 4.** Clustering of biological process enrichment analysis.


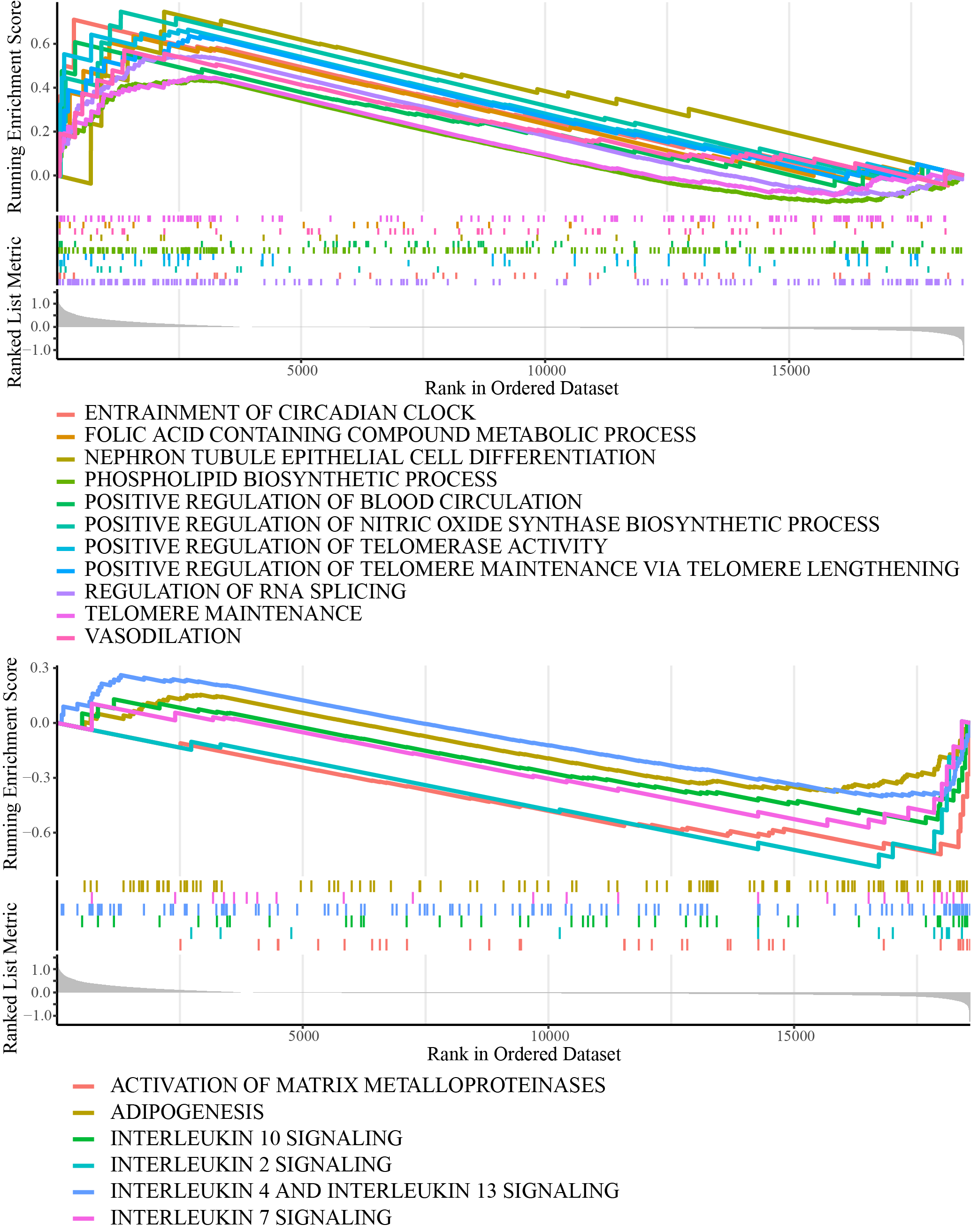


**Supplementary Figure 5.** GSEA enrichment analysis of transcriptome before and after high lutein intake.

**
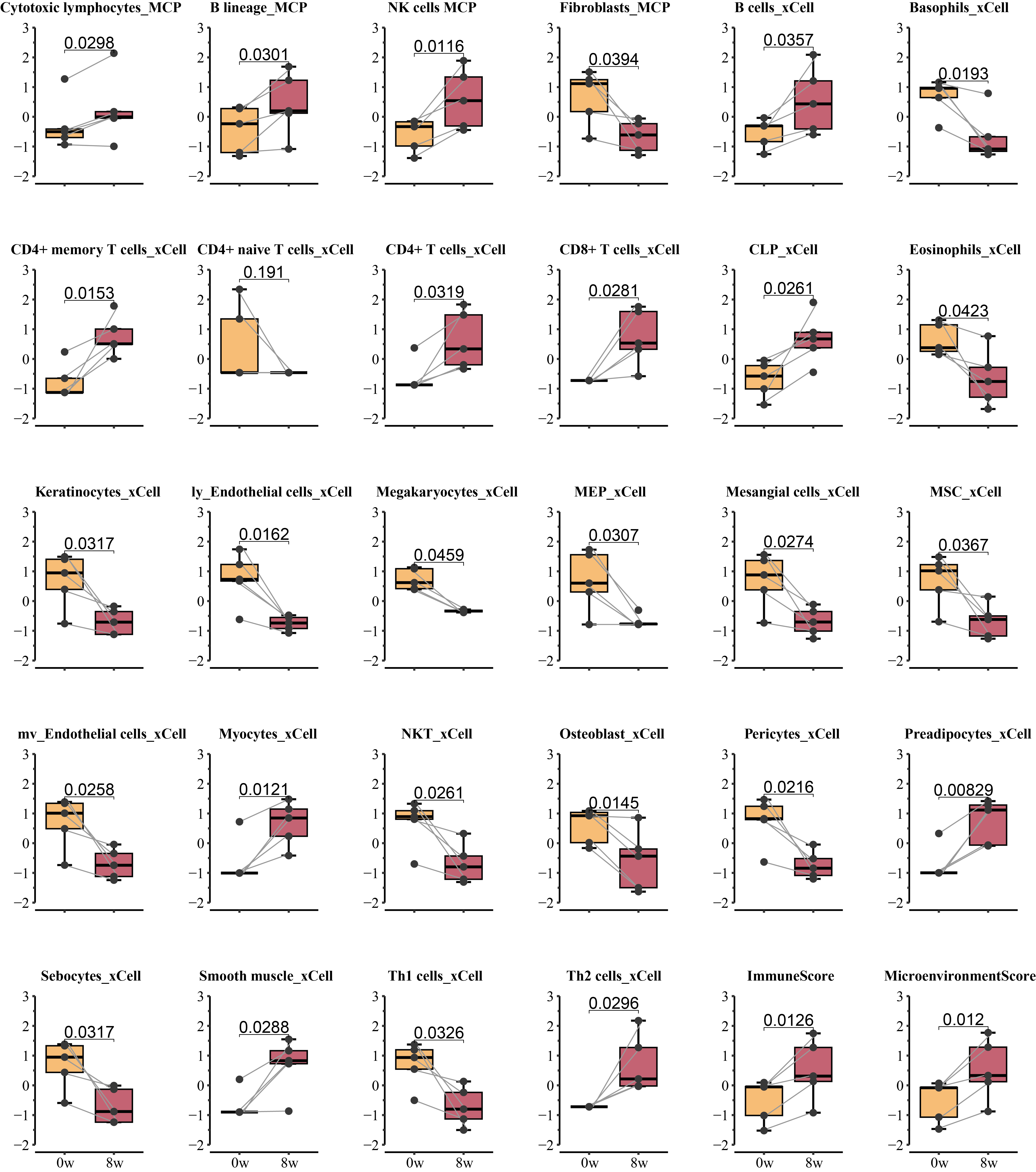
**

**Supplementary Figure 6.** Difference of immune abundance before and after high lutein intake.

**
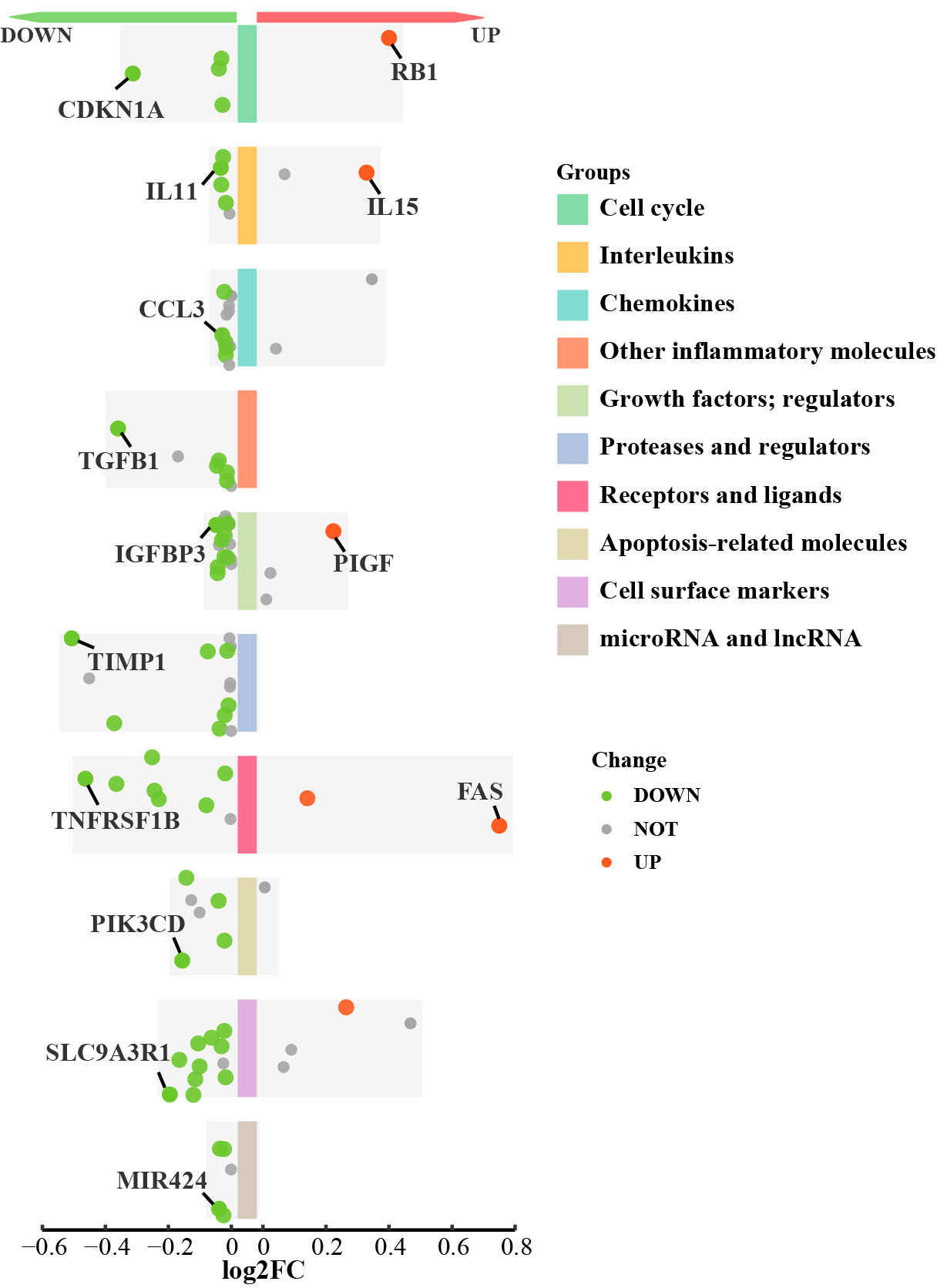
**

**Supplementary Figure 7.** Transcriptome expression difference of aging-related marker before and after high lutein intake. The genes with the most significant differences in expression in both directions were marked in each group.
